# Towards equitable carbon responsibility: Integrating trade-related emissions and carbon sinks in urban decarbonization

**DOI:** 10.1016/j.ese.2025.100539

**Published:** 2025-02-02

**Authors:** Junliang Wu, Yafei Wang, Shuya Zhang, Yu Zhu, Bingyue Fu, Zhihui Zhang, Hanxi Chen, Shaoqing Chen

**Affiliations:** a School of Environmental Science and Engineering, Sun Yat-sen University, Guangzhou, 510006, China; b Guangdong Provincial Key Laboratory of Environmental Pollution Control and Remediation Technology (Sun Yat-Sen University), Guangzhou, 510006, China; c Institute of Carbon Neutrality and Green Development (Sun Yat-sen University), Guangzhou, 510275, China

**Keywords:** Carbon mitigation responsibility, Carbon leakage, Forest carbon sink, Allocation framework, Guangdong–Hong Kong–Macao greater Bay area

## Abstract

Cities play a pivotal role in global decarbonization, acting as a critical driver of carbon emissions. Accurately allocating carbon mitigation responsibility (CMR) is essential for designing effective and equitable climate policies. How cities manage carbon leakage across boundaries through supply chains and implement plan of increasing forest carbon sinks are important components for designing a fair and inclusive CMR. However, the combined impact of trade-related carbon leakage and forest carbon sinks on CMR allocation remains poorly understood. Here, we develop an integrated CMR allocation framework that accounts for both carbon leakage and variation of forest carbon offsets. When applied to the cities within the Guangdong–Hong Kong–Macao Greater Bay Area in China, it becomes evident that the inclusion of carbon leakage results in substantial alterations in mitigation quotas. Adjustments are observed to vary between ±10 % and 50 % across these cities from 2005 to 2020, a trend that is anticipated to continue until 2035. The redistribution of outsourced emissions through supply chains alleviates the mitigation burden on producer cities by 20–30 %. Additionally, accounting for carbon sinks substantially influences CMR allocation, particularly in forest-rich cities, which may see their carbon budgets increase by up to 10 %. Under an enhanced climate policy scenario, the growth rate of total mitigation quotas from 2025 to 2035 is projected to decrease by 40 % compared to a business-as-usual trajectory, reducing the burden on major producer cities. Our proposed CMR framework provides a robust basis for incentivizing coordinated mitigation efforts, promoting decarbonization in supply chains and enhancement of urban carbon sink capacities.

## Highlights

• Incorporating carbon leakage into mitigation strategies can alter cities' quotas by ±10 %–50 % in GBA cities.
• Accounting for carbon sinks increases carbon budgets by up to 10 % in cities with significant forest areas.
• The mitigation burden on producer cities could be reduced by 20–30 % through redistributing outsourced emissions.
• This framework encourages equitable and coordinated decarbonization by addressing carbon leakage and sinks variation.

## Graphical abstract

**Figure:**
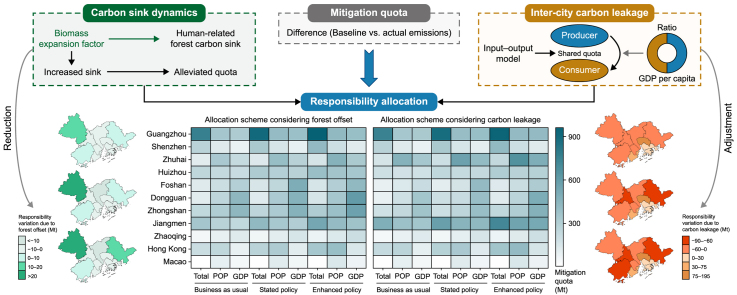

## 1 Introduction

Global climate change has become one of the most severe environmental challenges today [1,2], necessitating tremendous efforts to limit global temperature rise within 1.5–2.0 °C following the Paris Agreement signed by many nations [[3], [4], [5]]. As key areas in combating climate change [6,7], many cities have proposed carbon neutrality targets with timelines spanning from 2035 to 2060, mostly aiming to reduce anthropogenic CO_2_ emissions and increase sinks and removals [[8], [9], [10]]. Related to this, it is crucial to ensure efficient and equitable mechanisms for determining emission quotas and mitigation responsibilities, which, in turn, foster coordinated climate actions at local levels [11,12]. Although cities are vibrant hubs of trade activities that are indispensable in driving land-use changes and motivating regional and global supply chains, they also pose challenges related to carbon leakage [13,14]. These factors increase the complexity and uncertainty of determining the carbon mitigation responsibility (CMR) shared by cities.

Current CMR allocation typically aims to balance equity and efficiency, particularly considering various factors, such as emission scale, economic capability, technology capabilities, and human rights [[15], [16], [17]]. Studies have also shown that the allocation of emission quotas should consider inclusiveness across regions of different development statuses, considering socioeconomic factors, technology, and carbon dioxide removal capability [[18], [19], [20]]. Thus, based on capability and historical emissions principles, cities with great emission scopes or higher consumption levels, such as Beijing and Tokyo, must shoulder greater responsibilities compared to cities with lower economic outputs and less reliance on imported goods, such as Tianjin and Chongqing [18,[21], [22], [23]].

Redistributing “shared responsibilities” through supply chains has also attracted wide attention [[24], [25], [26]], with several studies exploring the phenomenon of responsibility transfer due to trade-related carbon leakage [[27], [28], [29], [30]]. Other studies have recommended that over 20 % of carbon responsibility be reallocated throughout the global supply chain by addressing the carbon leakage issue between producing and consuming hubs [31,32]. After factoring in economic welfare and trade-related carbon transfers, regions with higher economic affluence are allocated greater responsibilities, thus underscoring the importance of incorporating the production and consumption perspectives into the study of responsibility allocation [33]. For example, regional CMR studies showed that more developed regions in China, such as Guangdong Province, were projected to have larger carbon emission quotas by 2030 compared to other regions, such as Xinjiang and Nei Mongol, because of the former's bigger economic scale, stronger industrial activities, and greater energy consumption, all of which necessitate larger carbon emission quotas to sustain economic growth in these regions [34,35]. In Guangdong, central cities such as Guangzhou and Shenzhen receive higher quota allocations under the “intensity” principle, whereas quotas for surrounding cities tend to increase under the per capita principle [36,37]. While other studies have assessed land use and forest management as part of cities' efforts to achieve decarbonization, changes in carbon sinks have not been reflected in the CMR allocation scheme.

To achieve the carbon neutrality target in cities, the schemes for implementing CMR should adopt insights from accounts of carbon source and sink, which can be considered along with equity, efficiency, and inclusiveness. First, under the common but differentiated responsibilities (CBDR) principle proposed by the United Nations Framework Convention on Climate Change (UNFCCC) [38], consumers in well-developed regions should bear the responsibility of carbon mitigation, even though emissions occur beyond their territorial boundaries. Quantifying the effects of trade-related carbon leakage and integrating them into the CMR allocation scheme can lead to establishing a more inclusive scheme that can potentially address the inherent dilemma brought about by regional heterogeneity and rivalries [[39], [40], [41]].

Second, changes in urban land use and forestation could also be reflected in CMR, as these are currently trackable and quantifiable human endeavors of carbon offsetting. With better ecosystem conservation and forest management, natural carbon sinks can neutralize significant carbon emissions, thus offering a powerful, nature-based solution for climate change mitigation [[42], [43], [44]]. Therefore, variations in forest carbon sinks, particularly those with trading potential, should be considered when adjusting future CMR allocations among cities on a comparative basis [[45], [46], [47]]. Incorporating carbon leakages and sinks into the design of a CMR scheme helps address the need to give credit to cities already achieving progress in their carbon mitigation and offsetting efforts, which are equally important in achieving carbon neutrality. However, the effectiveness of such a scheme has yet to be tested in cities closely linked by trade, such as those within urban agglomeration. The impact of incorporating carbon leakage and sink variations into identifying intercity CMR should be regularly tracked over time, thereby underscoring the diversified roles cities play in advancing decarbonization.

To address this research gap in the current study, we developed a new CMR allocation scheme among cities, which includes intercity carbon leakage and variations in forest carbon offset in the mechanism of mitigation responsibility sharing. This goal is achieved from two perspectives, namely, production and consumption, while also considering various socioeconomic variables, such as the urban population shares and gross domestic product (GDP). Using all 11 cities in the Guangdong–Hong Kong–Macao Greater Bay Area (GBA), one of the most populated urban agglomerations worldwide, as a case study, we calculate the CMR allocation based on inter-regional carbon flows and changes of forest offset between 2005 and 2020 from the total, per capita, and intensity-based accounts. As a highly populated and economically diverse region, the GBA includes cities with distinct economic structures and varied political systems. Such diversity, along with the active trade flows and abundant forest resources in the region, makes the GBA an ideal test case for CMR schemes that consider interregional carbon leakage and forest carbon sinks. The CMR is traced along global and regional supply chains and then distributed to each city based on the established scheme. Next, we project future changes in CMR until 2035 using the results of a scenario-based simulation of cities’ diverging socioeconomic development and carbon emission trajectories. By doing so, we demonstrate the merits of the proposed CMR scheme in stimulating comparable and equitable decarbonization actions among cities closely linked through their supply chains.

## 2 Methodology

### 2.1 CMR assessment framework

**Fig. 1:**
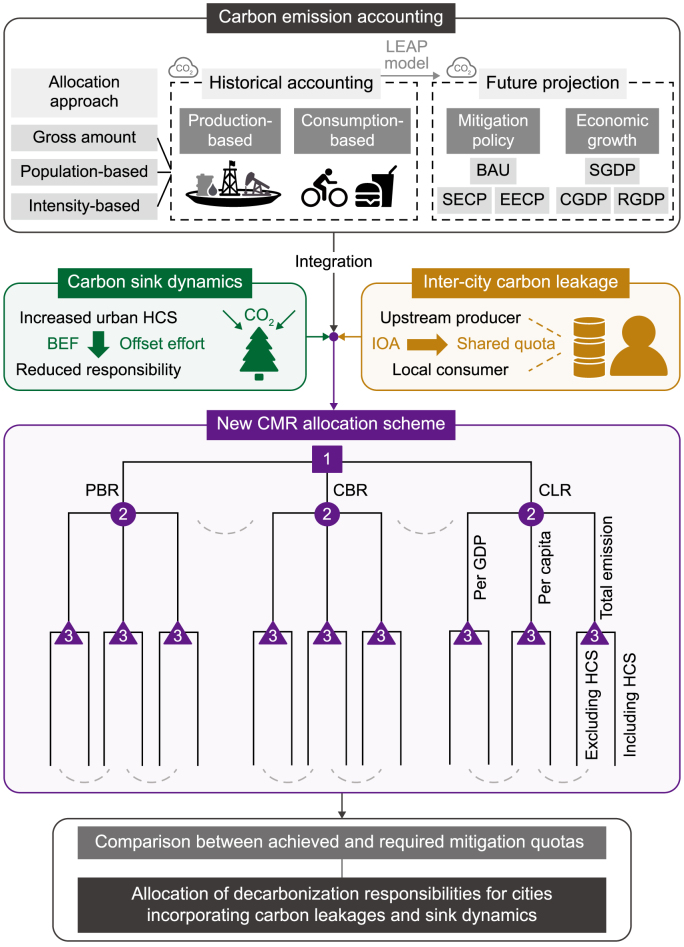
Carbon mitigation responsibility (CMR) allocation scheme applied to cities by incorporating trade-related carbon leakages and carbon sink dynamics. LEAP: Long Range Energy Alternatives Planning System; BAU: business-as-usual; SECP: stated energy and climate policy scenario; EECP: enhanced energy and climate policy scenario; GDP: gross domestic product; CGDP: conservative GDP growth; SGDP: steady GDP growth; RGDP: robust GDP growth; HCS: human-related forest carbon sink; BEF: biomass expansion factor; IOA: input–output analysis; PBR: production-based carbon mitigation responsibility; CBR: consumption-based carbon mitigation responsibility; CLR: carbon mitigation responsibility considering the effects of trade-related carbon leakages.

In this study, we developed a CMR allocation scheme considering multiple socioeconomic factors from the production- and consumption-based perspectives (Fig. 1). Initially, we determined the basic CMR of the 11 selected cities based on emissions represented by gross amount, per capita, and intensity. Employing input–output analysis (IOA), the carbon leakages between upstream producers and local consumers were accounted for based on the CBDR principle. At the same time, the human–related forest carbon sink (HCS) was quantified using the biomass expansion factor (BEF) method, wherein every city's offset effort contribution to CMR was evaluated. Next, we forecasted the carbon emission trajectories of the cities using the long-range energy alternatives planning system (LEAP) model, in which a set of policy scenarios representing different paces of economic development and decarbonization were developed to simulate future CMR changes over time and across cities. By incorporating variations in carbon accounts and considering the relevant socioeconomic factors, we constructed 18 CMR allocation methods featuring trade-related carbon leakage and variations in forest carbon sinks. This allocation scheme assessed historical differences in CMR distribution and projected future changes in cities' CMR. Finally, we integrated population size and economic level into the CMR. Then we compared the responsibilities of cities based on the principles of total emission, per capita emission, and emission intensity. Through this process, we demonstrated how different socioeconomic factors influence CMR allocation from the production- and consumption-based perspectives.

### 2.2 City-scale carbon emission accounting

Here, we first assessed production-based carbon emissions (PBE), which are direct carbon emissions from cities [48,49]. Considering a comparable dataset for measuring the CMR of all cities, we focused on energy-related carbon emissions in this study. Consistent with the established Intergovernmental Panel on Climate Change (IPCC) guidelines [50], PBE can be calculated using equation (1):

(1) $$PBE=\sum_{k=1}^{}\sum_{q=1}^{}D_{q}^{k}\varepsilon_{q}O_{q}^{k}$$

where *q* represents energy types; *k* represents economic sectors [51]; *D* denotes activity data, here referring to energy consumption volume; and ε, and *O* represent the emission factor and oxygenation efficiency for each energy type during fuel combustion, respectively [52,53].

Next, consumption-based carbon emissions (CBE) were estimated using multiple-regional input–output (MRIO) analysis, a commonly utilized approach across various fields of study [[54], [55], [56]]. A nested MRIO table was constructed by connecting Chinese MRIO with global MRIO to capture “inter-regional carbon leakage,” defined in this study as the transfer of carbon emissions across borders. Detailed explanations of establishing a nested MRIO table are shown in Fig. S1 (Supplementary Material). Specifically, “net importer” produces locally but consumes elsewhere, while “net exporter” sends emissions to other regions. Their respective calculations are shown in equations (2), (3). A detailed accounting process is provided in Text S1 (Supplementary Material). The formulas for equations (2), (3) are as follows:

(2) $$CBE=\left[e_{CO_{2}}\right]\times L\times Y$$

(3) $${CF}_{\text{net}}={CF}_{\text{in}}-{CF}_{\text{out}}$$

where $\left[e_{CO_{2}}\right]$ is the vector of carbon emission intensities of sectors; $L={\left(I-A\right)}^{-1}$ represents the Leontief inverse matrix, with A and I denoting the direct technical coefficient matrix and the identity matrix, respectively; *Y* represents the final demand matrix; ${CF}_{\text{net}}$ represents the net carbon inflows; and ${CF}_{\text{in}}$ and ${CF}_{\text{out}}$ denote carbon inflows and outflows, respectively.

Net carbon emissions were calculated by adjusting the total emissions (depending on whether they were production- or consumption-based). This was done by incorporating the effects of HCS and trade-related carbon leakage (NCL). Forest carbon offsets reduce emissions through carbon sequestration in human-related urban forests, while trade-related carbon leakage accounts for emissions transferred between regions due to economic activities. The responsibility for these transferred emissions is shared between producer and consumer regions based on their economic capacities, measured by the GDP per capita. This calculation system ensures a fair distribution of carbon leakage responsibility. The final net carbon emissions for production-based emissions (NPE) or consumption-based emissions (NCE) were calculated using equations (4), (5), respectively, factoring in forest offsets and trade-related carbon leakage. In the case of the GBA, while its total consumption-based carbon emissions are influenced by trade within and outside the region, our analysis focuses on carbon leakage at the intercity level within the GBA. By doing so, we targeted mitigation quota distribution among the GBA cities and policies of urban decarbonization, which can be implemented within administrative boundaries. Detailed calculations for sharing carbon leakage responsibilities are provided in Text S2 (Supplementary Material).

In this study, we defined “net carbon emission” based on whether it refers to production- or consumption-based emissions, considering the effects of forest carbon offset and trade-related carbon leakage. Forest carbon offset accounts for carbon reduction through natural sequestration, while trade-related carbon leakage represents the emissions transferred between regions due to consumer demand. The responsibility for these transferred emissions is divided between producer and consumer regions based on their economic capacities, as measured by their GDP per capita, thus leading to a fair distribution of carbon leakage responsibility. The NPE and NCE were calculated using equations (4), (5):

(4) NPE=PBE−HCS+NCL

(5) NCE=CBE−HCS−NCL

where NPE and NCE are net production- and consumption-based carbon emissions factoring in forest offset and carbon leakage, respectively, and NCL is the net trade-related carbon leakage from a city to other regions. The responsibility for leakages is jointly shouldered by the producer and consumer based on their economic capabilities. The detailed method of how carbon leakage responsibility is shared between producer and consumer cities is described in Text S2 (Supplementary Material).

Based on the information presented above, we projected the future PBE and CBE of the GBA and its cities using the LEAP model, widely applied in forecasting energy demand, consumption, and environmental impacts in various sectors [57,58]. In particular, we built the LEAP model for GBA cities based on data on energy consumption and socioeconomic factors. The LEAP model was then integrated with an emission method to estimate carbon emissions from top to bottom. Detailed descriptions and equations of the method are presented in Text S3 (Supplementary Material).

### 2.3 Accounting for forest carbon sinks

In this study, we considered changes in carbon sinks as an important component of the CMR allocation scheme. In particular, we accounted for the increase or decrease in HCS in the cities and assessed its impact on their corresponding mitigation responsibilities. For example, larger carbon budgets can be afforded for cities with abundant forest resources. Here, HCS represents the potential for tradable carbon sinks related to human activities, that is, the carbon sequestration potential of existing young and middle-aged forests. We identified and calculated the HCS for tradable carbon offsets in cities based on the BEF approach, including artificial and natural young and middle-aged forests. The estimation of HCS follows equation (6) with a detailed description of the computation provided in Text S4 (Supplementary Material).

(6) $$HCS=\sum_{i=1}^{n}\sum_{j=1}^{m}\left[A_{ij}\times V_{ij}\times R_{ij}\times D_{ij}\times {BEF}_{ij}\times \left(1+B_{ij}\right)\times {CF}_{ij}\right]$$

In equation (6), A represents the areas of young and middle-aged arbor forests; V represents the stock volume per unit area; R denotes the growth rate of stock volume; D is the basic timber density; BEF represents the biomass expansion factor; B represents the proportion of below-ground biomass and above-ground biomass; CF refers to the carbon content rate of biomass; i represents cities; n denotes the number of cities, here referring to the 11 cities; j refers to the arbor species; and m denotes the number of species, here referring to the two species. To calculate the annual forest carbon sinks among the cities, interpolation and extrapolation methods (Supplementary Material Fig. S2) were applied.

### 2.4 Measurement of intercity CMR

The CMR allocation scheme developed in this study covers intensity reduction efforts, socioeconomic development, and direct and indirect carbon emissions from urban activities. The total responsibility or mitigation quota was quantified by comparing the baseline emissions (based on constant carbon intensity) with actual emissions during a specific period. This step provides a framework to estimate the impact of sustained efforts and to plan necessary strategies to achieve carbon peaking. Specifically, mitigation responsibility is represented through various types of mitigation quotas.

In this work, we distinguish between the achieved mitigation quota (AMQ) and the required mitigation quota (RMQ) to evaluate the mitigation efforts of cities. In particular, AMQ refers to the emission reduction already realized by a city. “Future mitigation quota” refers to the reduction the GBA is expected to achieve within a specified period based on carbon reduction scenarios and economic growth. The proportion of AMQ to baseline emission represents the various internal efforts exerted to achieve emissions reduction and economic development. RMQ is determined by distributing the overall national or regional mitigation quota to each city. The difference between RMQ and AMQ, also known as the mitigation quota difference (MD), was used to assess cities’ capabilities in fulfilling their respective responsibilities. The mitigation quota of each city is quantified using equations (7), (8), (9), (10), (11):

(7) $$AMQ=e_{t_{0}}\times {GDP}_{t_{1}}-{CE}_{t_{1}}\text{,}$$

(8) $${RMQ}_{i}=AMQ\times {QS}_{i}\text{,}$$

(9) $${QS}_{i}=\frac{{CE}_{i}}{\sum_{i=1}^{n}{CE}_{i}}\text{,}$$

(10) $${AMQ}_{i}=e_{t_{0},i}\times {GDP}_{t_{1},i}-{CE}_{t_{1},i}\text{,}$$

(11) $${MD}_{i}={RMQ}_{i}-{AMQ}_{i}\text{,}$$

where ${CE}_{i}$ denotes the carbon emission of city i under various principles; $t_{0}$ and $t_{1}$ represent the baseline and final years, respectively; $e_{t_{0}}$ is the carbon intensity (carbon emission per unit of GDP) of the baseline year; ${GDP}_{t_{1}}$ denotes the urban gross domestic product of the final year; and ${RMQ}_{i}$*,* ${AMQ}_{i}$, and ${QS}_{i}$ denote the RMQ, AMQ, and quota share of city *i*, respectively. Based on each city's reduction targets, the GBA's overall mitigation quota was calculated by incorporating projected carbon emissions across various scenarios. Furthermore, using the carbon intensity in the baseline year as the benchmark, the future mitigation quota was estimated based on the anticipated economic growth rates and carbon intensity reduction goals. Each scenario produces distinct emissions, resulting in certain AMQ values. Building on the mitigation goals of the cities, the RMQ for each city was then determined by distributing the overall quota following each city's quota share ${QS}_{i}$ based on projected emissions. The allocation of total emissions was further adjusted by factoring in GDP, population, and carbon intensity, thus offering different accounting bases for distributing CMR among cities.

### 2.5 Scenario setting

Here, we set nine integrated policy scenarios considering population, economic growth, and energy transition. The three low-carbon policy scenarios were as follows: business-as-usual scenario considering the current trend of economic development and energy consumption (BAU); standard energy conservation policy considering timely carbon peaking and current planning goals, as outlined in the 14th Five-Year Plan (SECP); and enhanced energy policy considering advanced energy transition policies and technologies compared to existing plans (EECP). Meanwhile, the three economic growth scenarios included conservative GDP growth considering moderate economic growth strategies (CGDP); steady GDP growth considering planned economic trajectories (SGDP); and robust GDP growth considering accelerated economic expansion and higher growth rates (RGDP). The nine integrated policy scenarios were developed based on the simulation of future energy development pathways and economic growth in the GBA cities. The baseline year of the future simulation was set at 2020.

In projecting future carbon flows and leakages during 2020–2035, we assumed that the Leontief matrix remained constant relative to the baseline year. Based on the forecasted variations in carbon intensities and consumption levels across the nine scenarios, we updated the emission intensities and sectoral consumption in 2025, 2030, and 2035 by referring to the variations found in different scenarios. We used BEF to project the forest stock volume change rate based on local forestry development plans to estimate each city's carbon sink growth and offset potential. We assumed these values remained consistent with historical baseline levels for key parameters, such as wood density and biomass carbon content. For the years beyond the 14th Five-Year Plan (2021–2025) and the 15th Five-Year Plan (2026–2030) periods, our projections relied on consistent forest protection and management practices observed across the region, as well as on the policy continuity reflected in recent forestry and ecological conservation plans. This methodology allowed us to integrate planned and projected growth rates effectively, thus providing a comparative basis for estimating HCS potential across different cities. The details are described in Text S5 (Supplementary Material). The setting of the scenarios is described in Tables S1 and S2 (Supplementary Materials).

### 2.6 Case study and data

The GBA comprises nine populated cities in Guangdong Province and two special administrative regions of China (Hong Kong and Macao). As a major urban agglomeration, the GBA is the frontier of China's pursuit of high-quality and low-carbon economic development. This area boasts a GDP of approximately US$1820 billion and a population of about 67.7 million, with an overall urbanization rate exceeding 85 %. The GBA demonstrates significant economic diversity among cities, including variations in development stage and economic structure, which range from manufacturing centers to service-oriented economies. Furthermore, forest resources are unevenly distributed across the GBA cities, which means that these cities have made different efforts to increase their carbon sinks. As such, the GBA can well-demonstrate the process of developing and applying a new CMR allocation scheme at the urban agglomeration or regional scales, while incorporating trade-related carbon leakages and sink variation. A detailed description of the case study is provided in Text S6 (Supplementary Material).

To ensure consistency and comparability, socioeconomic data for each GBA city, including population, GDP, and energy consumption, were derived from official government statistical reports and relevant statistical yearbooks for individual cities and Guangdong Province. Original Chinese MRIO tables for 2007, 2012, and 2017, with a coverage of 31 provinces, were derived from past works [[59], [60], [61]]. Global MRIO datasets covering 189 countries and 4915 sectors were obtained from the Eora database. Key data on local carbon emissions for mainland China were sourced from the China Emission Accounts and Datasets (CEADs) database [[62], [63], [64]], while the data for other regions were supplemented by the PRIMAPHIST database in Eora [65,66]. Forest data, including the sizes and storage volumes of young and middle-aged arbor forests, were collected from the 6th to 9th National Forest Resources Inventory Bulletins of China [67]. Detailed descriptions of the data used are provided in Table S3 (Supplementary Material).

## 3 Results and discussion

### 3.1 Carbon emissions, sinks, and trade-related leakage

**Fig. 2:**
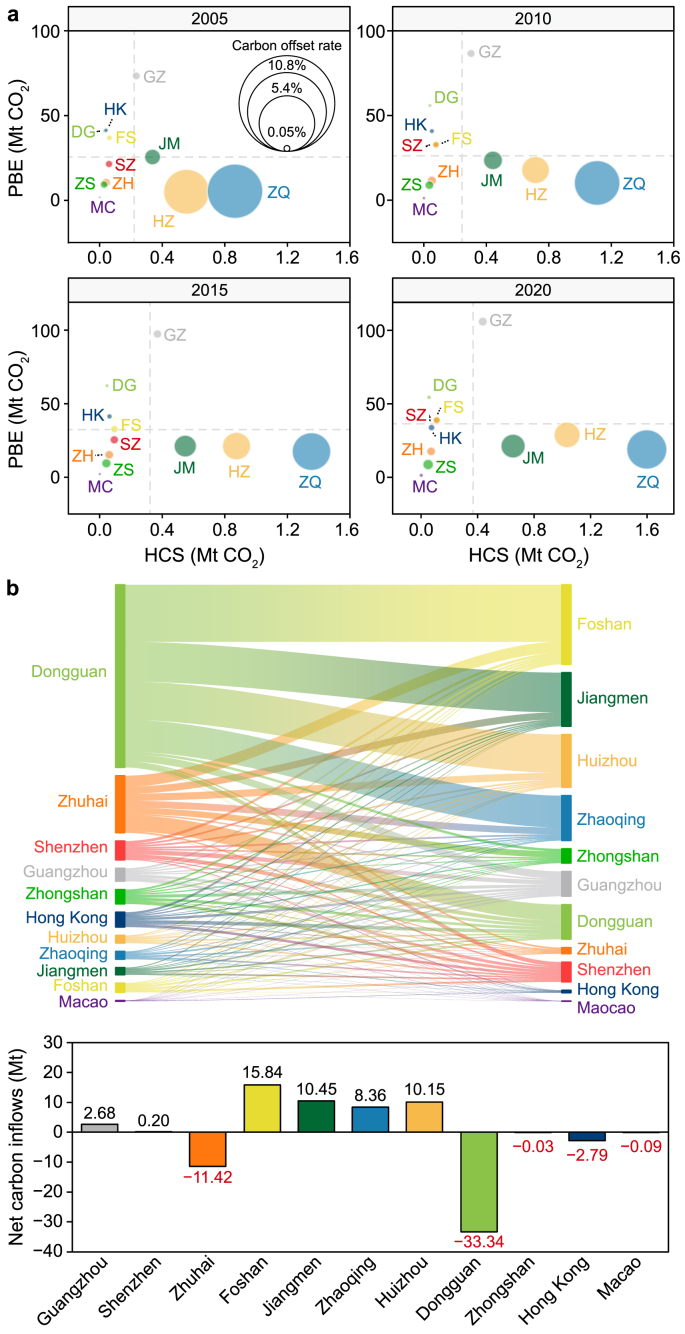
Dynamic distributions of production-based CO_2_ emissions (PBE) and human-related forest carbon sink (HCS) during 2005–2020 with trade-related carbon leakage. **a**, The relations between carbon emissions and forest carbon sinks across the cities. **b**, Trade-related carbon leakage among the cities of GBA in 2020. Cities' abbreviations: Guangzhou (GZ), Shenzhen (SZ), Huizhou (HZ), Jiangmen (JM), Foshan (FS), Zhaoqing (ZQ), Zhongshan (ZS), Zhuhai (ZH), Dongguan (DG), Hong Kong (HK), and Macao (MC).

The distribution and dynamics of the PBE, carbon sinks, and intercity carbon flows in the GBA are shown in Fig. 2a. From 2005 to 2020, the PBE of the GBA as a whole increased by 36 %, with Guangzhou and Dongguan being the two largest contributors. From a consumption-based perspective (Supplementary Material Fig. S3), Hong Kong leads in per capita emissions, followed by Shenzhen and Guangzhou. Over time, the disparity between high-emitting cities and others diminished due to accelerating economic growth and increasing consumption demand. However, a notable heterogeneity of forest carbon offset remains across the 11 cities of the GBA. Specifically, Zhaoqing, Huizhou, and Jiangmen show the highest carbon offsetting potentials due to their abundant forest resources and prevalent natural ecosystems [[68], [69], [70]]. The HCS in the GBA increased by 86 % between 2005 and 2020, largely driven by the expansion of plantations and enhanced forest conservation efforts [71,72]. Conserving natural forests in the GBA has significantly exceeded forests in increasing offset potential (Supplementary Material Fig. S4). However, a major challenge is that, over time, the carbon offset rate from either consumption-based or production-based accounts decreased in most cities, indicating that the growth of sinks has been lacking despite the increase in emissions.

Trade-related carbon leakages among the 11 cities of the GBA are shown in Fig. 2b, while the associated carbon flows between the GBA and the rest of the world are presented in Fig. S5 (Supplementary Material). Overall, the GBA has a relatively low impact on the transfer of carbon leakage responsibility to other regions outside China through its local consumption, amounting to only about 3 % of the responsibility allocated from abroad. When leakage responsibility is designated based on economic capability, developed countries, such as the United States (US) and European Union (EU) nations, account for a significant portion of the carbon leakages between them and the GBA, with the US accountable by 73 % and the EU nations by 60 %. Due to their intensive manufacturing activities, many cities in the GBA, such as Foshan and Jiangmen, are net emission importers (with 15.8 and 10.5 Mt imported emissions, respectively). In contrast, cities such as Hong Kong, Macao, and Zhuhai are considered key exporters. For example, Zhuhai is a major net emission exporter that has transferred over 10 Mt of carbon emissions to neighboring cities annually, with ∼13 Mt outsourced specifically to Foshan. Intercity carbon leakages account for 41 % and 18 % of total exporters' and importers’ emissions from the production-based perspective and 12 % and 10 % from the consumption-based perspective, making it a highly influential factor in CMR allocation.

**Fig. 3:**
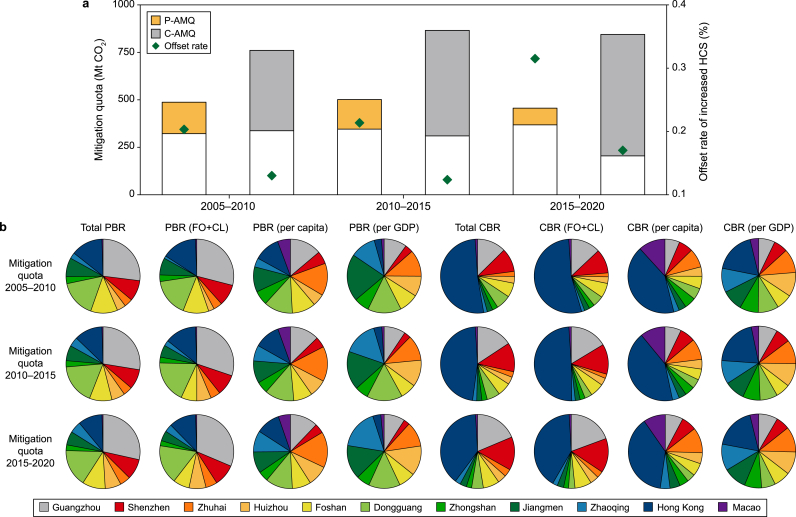
Historical carbon mitigation quota of Guangdong–Hong Kong–Macao Greater Bay Area (GBA) cities during the three historical periods (2005–2010, 2010–2015, and 2015–2020). **a**, Total historical achieved mitigation quota of GBA. **b**, Shares of required mitigation quota to GBA cities across various principles. P-AMQ and C-AMQ represent production- and consumption-based achieved mitigation quotas, respectively. HCS: human-related forest carbon sink; PBR: production-based carbon mitigation responsibility; CBR: consumption-based carbon mitigation responsibility. P-RMQ and C-RMQ represent production- and consumption-based required carbon mitigation quotas, respectively. FO: forest carbon offset; CL: carbon leakage. The white square with a black border represents the average annual actual emission.

The historical AMQs of the GBA from 2005 to 2020 from the production- and consumption-based perspectives are shown in Fig. 3a. For the GBA as a whole, the historical CMR and its proportion to baseline emissions decreased by 43 % and 47 % from 2010 to 2020, respectively. Further decreases could be more difficult despite the current reductions in the GBA cities’ carbon intensities. This trend will be affected by regional policies for industrial transfers and upgrading and enhanced environmental regulations [73,74].

Large variations exist in the allocation of RMQ across the GBA cities (Fig. 3b). Furthermore, the emission magnitude may differ significantly from the assigned responsibility. Mainland GBA cities with substantial manufacturing sectors have higher mitigation quotas under the production-based principle, mainly driven by local production activities [75]. Under consumption-based principles, the mitigation quotas of more developed cities tend to increase considerably. As indicated by our results, the RMQs in Hong Kong and Macao, whose emissions mainly originate from manufacturing hubs in northern and eastern China, notably increased by 270 % and 110 %, respectively [55,76]. Trade-related carbon leakage increased quotas of net exporters (consumers) by 7–12 % but decreased those of net importers (producers) by 20–50 % based on the production-based perspective. Under the intensity principle measured by emission per GDP, the mitigation quota assigned to Guangzhou could be reduced by ∼50 % from a production-based perspective, largely due to its service-oriented economy and cleaner energy usage [77,78]. Under the principle of wealthiness measured by GDP per capita [79,80], Hong Kong and Macao have notably larger shares (Supplementary Material Fig. S6). Meanwhile, under either the “per capita” or “intensity (GDP per emission)” principle, emissions from Huizhou, Zhongshan, and Zhaoqing have surpassed their RMQs.

The AMQs gradually surpassed the goals assigned to most cities. The MDs of Guangzhou, Jiangmen, and Zhuhai range from −7 to −20 Mt, with their actual carbon mitigation quotas exceeding their RMQs by 10–37 % under the “total emission” principle (Supplementary Material Fig. S7). For cities like Dongguan and Zhaoqing, their MDs transitioned from positive to negative from 2010 to 2020. After accounting for the joint effects of forest offset and carbon leakage, the MDs for most cities become smaller, with their achieved mitigation amounts becoming more matched with their RMQs. Among all the cities, Zhuhai struggled to meet its RMQ, highlighting the need for further strengthening its carbon management and reduction measures. Under the “per capita” and “intensity” principles, MDs in less developed cities (e.g., Zhaoqing, Jiangmen, and Zhongshan) are higher than zero, indicating that they are facing greater challenges in meeting their RMQs than others. Notably, under the “intensity” principle, the MDs of Hong Kong and Shenzhen range from −5 to −30 Mt, demonstrating their strong carbon reduction performance due to their fast-paced decarbonization driven by their stringent climate policies.

### 3.2 Future CMR allocated to cities

**Fig. 4:**
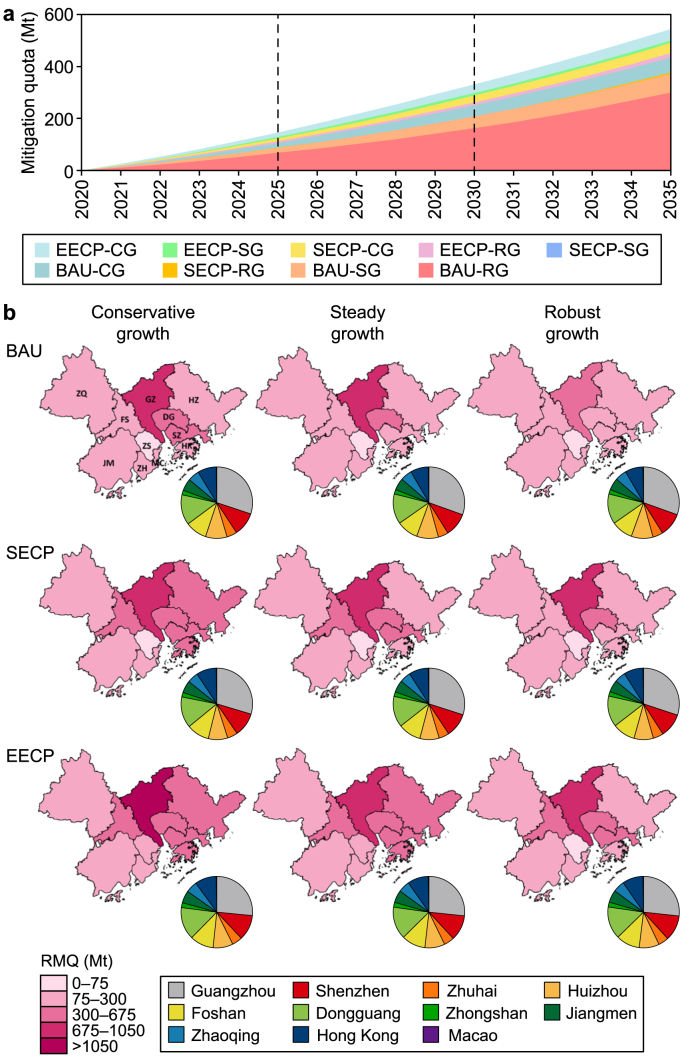
Future carbon mitigation quotas with the allocation of responsibilities during 2020–2035 under “total emission” principle across the nine scenarios. **a**, Projected production-based carbon mitigation quotas of Guangdong–Hong Kong–Macao Greater Bay Area (GBA), represented by the vertical distance from the top line of each scenario color area down to the *x*-axis. **b**, Future CMR allocation among cities from a production-based perspective. BAU: business-as-usual scenario; SECP: stated energy and climate policy scenario; EECP: enhanced energy and climate policy scenario; CG: conservative growth; SG: steady growth; RG: robust growth; RMQ: required mitigation quota.

The projected emissions and future CMR for the GBA and its cities are presented in Fig. 4a. Additional details can be found in Figs. S8 and S9 (Supplementary Materials), with further descriptions in Texts S7 and S8 (Supplementary Materials). Carbon emissions in the GBA are expected to peak before 2030 across most scenarios under the SECP and EECP, followed by a rapid reduction. The estimated cumulative mitigation quota for the GBA is projected to range from 2500 to 3000 Mt during 2020–2035, during which the values under the SECP scenarios are expected to be 19 % higher than those under the BAU scenario. This result means that stronger decarbonization measures are likely to increase the RMQs of the GBA cities. From 2020 to 2025, the GBA's mitigation quota under the EECP scenario is expected to be 51 % higher than that under the BAU scenario, although such a percentage is expected to decrease to 38 % by 2030–2035. The difference in future RMQs between the RGDP and CGDP scenarios is expected to narrow from 34 % to 20 % between 2025 and 2035.

The projected CMR for the 11 cities under the cap (total emission) principle during 2020–2035 can be found in Fig. 4b. Enhancing low-carbon measures under the EECP scenario can significantly improve mitigation quotas in Foshan, Hong Kong, and Shenzhen, with an increase of 60–70 % compared to the BAU scenario. In comparison, the cumulative mitigation quota of Guangzhou will remain at 600–1000 Mt during 2020–2035. Compared to a conservative pace of economic growth, the mitigation quota under RGDP will decrease by 50–60 % compared to the BAU scenario, while the mitigation quota will decrease by 25–30 % under the EECP scenario. Such values indicate that, on the one hand, the increase in mitigation quotas will be alleviated as low-carbon measures strengthen. On the other hand, the mitigation quotas of cities with large total emissions (e.g., Hong Kong and Shenzhen) will increase by 8–15 % under the EECP scenario compared to the BAU, thereby indicating the increased difficulty in balancing rapid economic growth and aggressive carbon reduction [81]. Over time, the spatial difference in mitigation quota distribution is expected to narrow (Supplementary Material Fig. S10). This phenomenon may be attributed to the faster RMQ growth trends of cities such as Huizhou, Dongguan, Zhaoqing, and Jiangmen compared to those of other cities. For example, in Huizhou, the mitigation quota shows a 5.0- to 6.5-times increase over 2020–2035, or an annual increase of about 17–21 % on average. In contrast, the growth of RMQs in more developed cities, such as Hong Kong and Macao, is likely to be slower, with an average annual increase of about 14–16 %.

**Fig. 5:**
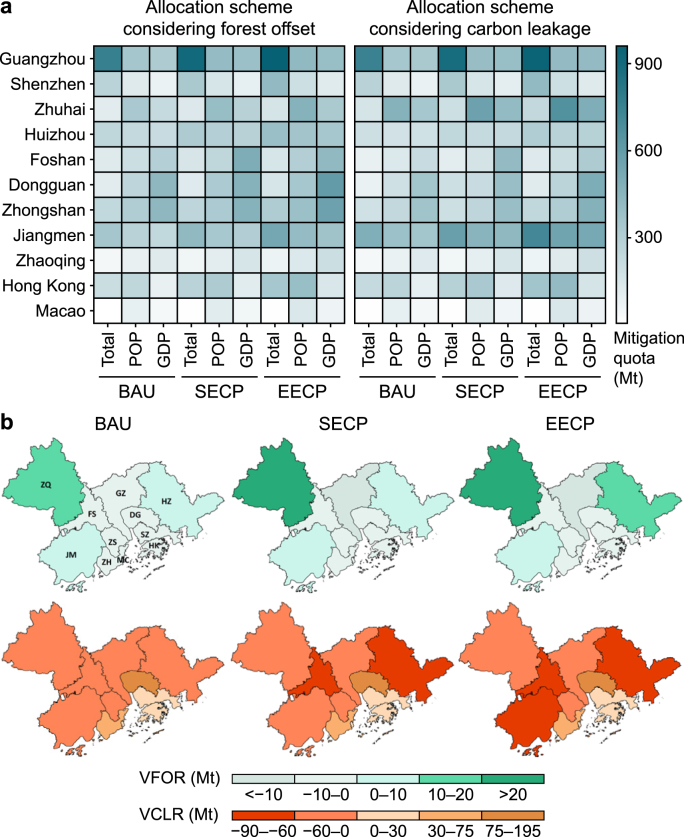
Future carbon mitigation responsibility (CMR) allocation of cities in Guangdong–Hong Kong–Macao Greater Bay Area (GBA) during 2020–2035 under steady GDP growth under business-as-usual scenario (BAU), stated energy and climate policy scenario (SECP), and enhanced energy and climate policy scenario (EECP). **a**, Future cumulative mitigation quotas during 2020–2035 considering socioeconomic factors among GBA cities. **b**, The effects of forest offset and carbon leakage on future CMR allocation. VFOR: the variation of responsibility allocation between considering and not considering the forest offset; VCLR: the variation of responsibility allocation between considering and not considering the trade-related carbon leakages.

The responsibility allocation from 2020 to 2035 considering forest offset and carbon leakage based on total emissions and socioeconomic factors is shown in Fig. 5a. Under the intensity measurement, higher mitigation quotas will be allocated to Jiangmen (12 %) and Zhaoqing (8 %), while the shares of Guangzhou and Shenzhen diminish. As the wealth disparity among the cities narrows, the mitigation responsibilities they shoulder will converge. Compared to the BAU scenario, the mitigation shares in most GBA cities are projected to increase by 3–10 % under the EECP scenario, while those of Huizhou and Zhaoqing are expected to remain unchanged. Cities could save on larger carbon budgets when their carbon reduction measures are strengthened.

The future effects of forest carbon offset and carbon leakage on responsibility allocation across the 11 cities are shown in Fig. 5b. The potential for expanding carbon sinks is significant, with a 6 % annual average increase of HCS (Supplementary Material Fig. S11), which leads to a 2–3% reduction in its RMQ during 2020–2035. In cities with abundant forest resources, such as Jiangmen, Zhaoqing, and Huizhou, the reduction in CMR could approach 5–10 % from 2020 to 2035. In comparison, the RMQs increased by 1.2–1.4 % in some cities with limited potential for HCS, such as Dongguan, Macao, and Hong Kong. Considering carbon leakage, the mitigation quotas of major carbon importers, such as Foshan, Huizhou, and Jiangmen, will be decreased by 20–30 % during 2020–2035. In comparison, the mitigation quotas of exporters, especially Dongguan and Zhuhai, will increase by 40–50 %. Increased HCS will mitigate mitigation quotas by around 4.5–9.0 Mt under the EECP scenario compared with BAU. Similarly, tracing back carbon leakage into consumers will also yield a CMR mitigation for producer cities by about 23 Mt during 2020–2035. To meet the carbon mitigation objectives, there should be increases in forest sinks (Supplementary Material Fig. S12) and reductions in carbon intensities (Supplementary Material Fig. S13). Furthermore, the offsetting potentials of cities such as Zhaoqing, Huizhou, and Jiangmen are expected to be enhanced with increased forest carbon sinks. In contrast, central cities, such as Shenzhen and Hong Kong, will continue to reduce carbon intensity.

Notably, the MDs of most cities are projected to be negative when considering the joint effects of forest sinks and carbon leakages. In particular, the cumulative MD of Dongguan is projected to reach a total of −500 Mt during 2020–2035 under the total emission principle, showing its great potential for proactive carbon mitigation (Supplementary Material Fig. S14). Developed cities, such as Hong Kong and Shenzhen, are expected to exceed their RMQs under the per capita and intensity principles, with their cumulative MDs ranging from −100 to −200 Mt. These values demonstrate that both cities' good carbon reduction performances are driven by their ambitious targets. Conversely, the MDs of Guangzhou and Foshan are projected to be 573 and 70.65 Mt, representing 175 % and 29 % of their RMQs, respectively. Such figures indicate that these cities' current energy and climate policies may be insufficient to meet their RMQs. In comparison, Macao is close to meeting its RMQ, with a difference representing only 0.15 % of its RMQ. Considering the effects of forest carbon sinks and carbon leakages, the MD of Foshan is reduced by nearly 90 %, while Guangzhou's will decrease by only 0.4 %. Therefore, more ambitious decarbonization targets and enhanced policy tools are essential for these cities to fulfill their mitigation responsibilities in the coming decades.

### 3.3 Policy implications of updating the CMR scheme

Addressing the requirements of inclusiveness, equity, and efficiency in responsibility allocation is crucial for carbon mitigation in cities, as these can reduce biases introduced by using individual indicators, such as the emission scale [79,82]. Given the notable difference in economic development, along with historical emissions, future CMR schemes should not only be adaptive and inclusive but must also recognize socioeconomic variations and the evolving dynamics of mitigation quotas. As such, it is important to determine the components capable of showing the mitigation efforts of individual cities under the CMR allocation scheme.

The CMR allocation scheme developed in the current study demonstrates strong adaptability with great potential for application across different spatial scales. This scheme is particularly useful for cities and regions with frequent trade flows, diverse geographical and economic characteristics, and significant carbon leakage challenges. The proposed allocation scheme targets the mitigation responsibilities of economies at various development stages, fostering coordinated carbon reduction strategies and localized development goals based on the reallocation of carbon emissions and forest resource endowments.

Based on this study's results, incorporating socioeconomic factors, forest carbon offsets, and trade-related flows can enhance fairness in responsibility distribution. Cities with greater efforts to increase their carbon offsets but have less carbon-intensive consumption should gain more carbon budgets. The selection of emission indicators is also vital. For example, cities that fulfill their assigned responsibilities under the per GDP and per capita principles but struggle with their “total emissions” should prioritize their total emission targets. This step is crucial for nations and cities that have carbon peak and neutrality goals on their course. The multifactor scheme can also help identify sufficiently effective but diversified mitigation pathways that suit the development needs of specific urban agglomerations and individual cities of different stages.

The proposed CMR allocation scheme distinguishes between emission, mitigation responsibility, and reduction capabilities, emphasizing strategic intercity collaborations and technology sharing to maximize regional mitigation efforts. This scheme also highlights the need for consumption-oriented cities (e.g., Hong Kong, Guangzhou, and Shenzhen) to ensure their outsourced industries in other regions adhere to stringent green standards. By doing so, these cities could play a much greater role in reducing emissions embedded in cross-regional supply chains—from an ethical or competent perspective. Although the underestimated responsibility of these consumer cities has been well recognized in the literature, policy tools to address this issue have been limited and constrained by complex cross-border negotiations [83,84]. Achieving this goal will require a systemic effort to evaluate responsibilities altered by carbon leakage.

Another equally interesting but less discussed issue is the treatment of forest carbon sinks. In this study, cities with richer forest resources, such as Zhaoqing, have an advantage in harvesting the potential for natural sequestration. However, caution must be applied in including this factor in formulating responsibility because the higher coverage of urban forests does not necessarily mean more prominent human-related increases in carbon sinks. Despite the challenges of delineating all influencing factors, the proposed CMR scheme could still be considered a first step in implementing responsibility allocation among cities closely connected by trade. Furthermore, the proposed scheme could provide targeted directions for policymakers involved in urban development planning, fostering regional cooperation in a just and low-carbon societal transition.

Another important feature of the proposed CMR scheme is that it highlights the need for inclusivity and adaptability over time. In particular, cities should update their mitigation responsibilities occasionally, depending on changes in developmental stages and decarbonization statuses [85]. Cities like Hong Kong and Guangzhou, which can achieve carbon peaking earlier, should undertake greater responsibilities in the early stages, providing more carbon budgets for less wealthy cities, such as Zhaoqing and Jiangmen, that are still catching up. However, these cities' RMQs could decrease once more advanced green technologies and cross-border tools are used to control their outsourced emissions. This strategy could also help decouple carbon emissions from other fast-developing cities' economic development and lower their mitigation quotas. In determining “when” and “where” the CMR should occur, incorporating multiple perspectives (cap, intensity, and per capita) is crucial in enabling a more comparable and ethical rule of decarbonization for cities to follow. Furthermore, promoting cross-regional carbon sink trading could be an effective market-oriented method for increasing emission mitigation for cities with larger carbon leakages and richer carbon sinks. In this regard, the timely update of the CMR scheme is crucial for ensuring cities’ efficiency and proactiveness as they implement local carbon mitigation plans tailored to their unique socioeconomic circumstances.

Robust financial mechanisms must be established to support the low-carbon transition of cities in the developing world. For example, leveraging green and climate bonds can secure long-term, low-cost funding for low-carbon infrastructure projects and renewable energy development. In addition, promoting technology diffusion could further enhance cities’ capabilities to adopt suitable environmental technologies, thereby ensuring reduced carbon mitigation costs while fostering economic growth. An intercity climate cooperation mechanism could also ensure that low-income, developing cities receive priority access to the green financing and technology support they need to meet their respective mitigation responsibilities. Furthermore, for cities with abundant tradable carbon sinks, implementing effective carbon market mechanisms can generate responsibility-driven carbon offset credits and generate income for local governments and residents.

### 3.4 Uncertainties and limitations

The carbon emission projections made in this study rely on key parameters, such as population, GDP, energy intensity, and energy structure. However, the primary sources of uncertainty arise from the assumptions about these parameters, especially over a long projection period. While these assumptions provide a reasonable basis, uncertainties remain due to potential variations in economic growth rates, energy consumption patterns, and carbon intensity improvements, especially because cities may deviate from their planned trajectories. For example, cities with large-scale industrial bases might experience unexpected energy intensities and emission shifts due to policy changes or economic restructuring. Furthermore, given the lack of official data extending to 2035, our projections are based on the “new normal” economic trend, which assumes a slower economic growth rate for China, in alignment with prior studies and observed historical trends. The uncertainties of future scenario projections highlight the importance of implementing flexible policy mechanisms, thus ensuring that mitigation responsibilities remain equitable and achievable despite differences in development paths.

## 4 Conclusions

Using 11 cities in the Guangdong–Hong Kong–Macao GBA as a case study, this study developed a novel CMR allocation scheme that integrated carbon leakages and variations of forest carbon offsets. The CMR scheme is constructed based on total, per capita, and intensity-based metrics from two perspectives: production and consumption. First, we determined the distribution of CMRs among cities from 2005 to 2020 based on different principles. Furthermore, we projected future changes in CMR until 2035 based on a set of policy scenarios, considering diverging socioeconomic development paths and carbon emission trajectories. The main findings of our study are as follows:

(1) Significant disparities exist in determining effective responsibility allocation between the production and consumption perspectives. In particular, Guangzhou and Dongguan have higher mitigation quotas on the production side, while Hong Kong and Macao have significantly higher quotas on the consumption side. Furthermore, reallocating carbon leakage responsibilities based on cities' economic capabilities reduces RMQs by more than 30 % in cities like Foshan and Zhaoqing. The potential for increasing forest carbon sinks is significant, with a 6 % annual increase of HCS, which translates to a 2–3 % reduction of its RMQ.
(2) As low-carbon measures strengthen (from the BAU to EECP scenarios), the RMQs for the GBA and most mainland cities will increase significantly. Over time, although these RMQs will continue to rise until 2035, their growth rates will decline. However, cities with large total historical emissions, such as Hong Kong and Shenzhen, will witness a rise in their RMQ shares, whereas Foshan and Jiangmen will experience reduced RMQs. The difference between AMQs and RMQs reveals that less developed and manufacturing-oriented cities, such as Zhongshan and Jiangmen, face greater challenges in meeting their RMQs than other cities.
(3) The proposed CMR allocation scheme is adaptive to the economics of different scales, thus recognizing socioeconomic differences and evolving mitigation needs. This scheme can stimulate cities' actions to better manage carbon leakage and increase human-related forest carbon sinks. On the one hand, developed cities with higher RMQs, strict green standards, and intercity collaborations can help reduce their emissions from the consumption side. On the other hand, developing cities with rich carbon sinks can benefit from incentives for enhancing sinks and regional carbon trading and prioritizing production-based measures to meet their mitigation responsibilities. Ultimately, aligning responsibilities with each city's role and carbon leakage dynamics could increase the built-in potential of urban decarbonization and foster regional cooperation in building a just and low-carbon society.

## CRediT authorship contribution statement

**Junliang Wu:** Writing – original draft, Methodology, Formal analysis. **Yafei Wang:** Writing – review & editing, Investigation, Formal analysis. **Shuya Zhang:** Investigation, Data curation. **Yu Zhu:** Visualization, Validation, Conceptualization. **Bingyue Fu:** Validation, Data curation. **Zhihui Zhang:** Visualization, Investigation. **Hanxi Chen:** Writing – review & editing, Validation. **Shaoqing Chen:** Writing – review & editing, Supervision, Funding acquisition, Conceptualization.

## Data availability

Data will be made available on request.

## Declaration of competing interest

The authors declare that they have no known competing financial interests or personal relationships that could have appeared to influence the work reported in this paper.
