# Supplementary material for: Towards equitable carbon responsibility: Integrating trade-related emissions and carbon sinks in urban decarbonization
Source: Environ Sci Ecotechnol. 2025 Feb 2;24:100539. doi: 10.1016/j.ese.2025.100539 (PMC11846929; doi:10.1016/j.ese.2025.100539)
Supplement: Multimedia component 1 [file mmc1.pdf]

Supplementary Information

Towards Equitable Carbon Responsibility: Integrating Trade-Related Emissions and Carbon Sinks in Urban Decarbonization

This file includes:

Supplementary Information

Pages S1 to S31

Text

Figure S1 to S14

Table S1 to S3

SI-Reference

Content

A. Supplementary Texts **3**

A.1 Accounting framework of consumption-based carbon emission.................................... 3

A.2 Ways of sharing carbon leakage responsibilities... 5

A.3 Detailed projects of carbon peaking pathway by LEAP model...................................... 6

A.4 Introduction of BEF and the estimation of HCS 7

A.5 Projects of future HCS... 9

A.6 Study area 10

A.7 Production-based carbon peaking pathways for the 11 cities of GBA 11

A.8 Consumption-based carbon predictions for GBA... 16

B. Supplementary Figures **17**

C. Supplementary Tables **27**

A. Supplementary Texts

Text S1 Accounting framework of consumption-based carbon emission

The basic equations for calculating consumption-based carbon emissions are as equations (A.1)–(A.7).

$$e_{\mathrm{CO}_2}{}_r^i = \frac{d_{\mathrm{CO}_2}{}_r^i}{X_r^i} \quad (A.1)$$

where $e_{CO_2}{}_i^r$ is the carbon emission intensity of region i and sector r; $d_{\mathrm{CO}_2}{}_r^i$ represents the direct carbon emission of region i and sector r; X_r^i represents total output of region i and sector r.

$$X = (I - A)^{-1} \times Y, \quad (A.2)$$

$$X = \begin{bmatrix} X_1 \\ X_2 \\ \vdots \\ X_{\mathrm{n}-1} \\ X_\mathrm{n} \end{bmatrix}, A = \begin{bmatrix} a_{11} & a_{12} & \cdots & a_{1\mathrm{n}} \\ a_{21} & a_{22} & & a_{2\mathrm{n}} \\ \vdots & & \ddots & \vdots \\ a_{\mathrm{n}1} & a_{\mathrm{n}2} & \cdots & a_{\mathrm{nn}} \end{bmatrix}, Y = \begin{bmatrix} y_{11} & y_{12} & \cdots & y_{1\mathrm{m}} \\ y_{21} & y_{22} & & y_{2\mathrm{m}} \\ \vdots & & \ddots & \vdots \\ y_{\mathrm{n}1} & y_{\mathrm{n}2} & \cdots & y_{\mathrm{nm}} \end{bmatrix}, \quad (A.3)$$

$$A = Z \times (diag(X))^{-1}, \quad (A.4)$$

$$Z = \begin{bmatrix} z_{11} & z_{12} & \cdots & z_{1\mathrm{n}} \\ z_{21} & z_{22} & & z_{2\mathrm{n}} \\ \vdots & & \ddots & \vdots \\ z_{\mathrm{n}1} & z_{\mathrm{n}2} & \cdots & z_{\mathrm{nn}} \end{bmatrix}, \quad (A.5)$$

where X, Y, I represent total output, final demand and identity matrix; $(I - A)^{-1}$ represents Leontief inverse matrix. The technical coefficient matrix $A = (a_{rs}^{ij})$ is obtained by $a_{rs}^{ij} = z_{rs}^{ij}/x_s^j$, in which z_{rs}^{ij} refers to the intersectoral monetary flows from sector r in region i to sector s in region j, and x_s^j is the total output of sector s in region j.

$$L = (I - A)^{-1}, A = [a_{rs}^{ij}] \quad (A.6)$$

$$[CBE]_{1\times\mathrm{m}} = [e_{CO_2}]_{1\times\mathrm{n}} \times L_{\mathrm{n}\times\mathrm{n}} \times Y_{\mathrm{n}\times\mathrm{m}} \quad (A.7)$$

where CBE represents consumption-based carbon emissions.

To estimate the CBE of respective cities, we assumed that the intersectoral monetary flows and carbon intensity within each sector for 2005, would replicate those in 2007, while then again, equivalently for 2010 and 2015 to emulate 2012, and for

2020 to reflect those of 2017. To align with consumption categories stipulated by MRIO tables, we first collected four consumption data of each city and assigned the items from these to different IO sectors and regions based on the proportions of values of final demand matrixes in nested MRIO tables. And then the row vectors of final consumption for the 11 cities were established.

In estimating carbon inflows and outflows, the equations can be regarded as equations (A.8) and (A.9).

$$C_{\text{in}} = \sum_{i=1} cf_{ij} \;;\; C_{\text{out}} = \sum_{j=1} cf_{ji} \tag{A.8}$$

$$C = \begin{pmatrix} cf_{ii} & cf_{ij} \\ cf_{ji} & cf_{jj} \end{pmatrix} = \begin{pmatrix} e_i & 0 \\ 0 & e_j \end{pmatrix} \begin{pmatrix} L_{ii} & L_{ij} \\ L_{ji} & L_{jj} \end{pmatrix} \begin{pmatrix} y_{ii} & y_{ij} \\ y_{ji} & y_{jj} \end{pmatrix} \tag{A.9}$$

where e_i and e_j are direct flow intensities of sectors in region i and region j; cf_{ij} denotes intra- and inter-regional carbon flows (between region i and region j), y_{ij} represents the final demand consumption of region j that is met by the production in region i.

Text S2 Ways of sharing carbon leakage responsibilities

In allocating the responsibility of trade-related carbon leakage between cities, we mainly focused on the emissions production produced by local region but consumed by other regions along regional supply chains. Following common but differentiated responsibilities (CBDR) principle, developed countries and regions are expected to take the lead in undertaking larger responsibility. Similarly, China's governors advocate for regions with the capability to prioritize achieving carbon reduction targets. In accordance with the concepts and ideas, the responsibility is assumed be shared both by producer and consumer based the ratio between their GDP per capita. For the carbon flows produced in region i but consumed in region j (cf_{ij}), the allocation for carbon leakage responsibility (CLR) is shown as equations (A.10) and (A.11).

$$CLR_{i(j)} = \varepsilon_{r(j)} \times cf_{ij}; CLR_{j(i)} = \varepsilon_{s(i)} \times cf_{ij} \quad (A.10)$$

$$\varepsilon_{i(j)} = \frac{GDP_i}{GDP_i+GDP_j}; \varepsilon_{j(i)} = \frac{GDP_j}{GDP_i+GDP_j} \quad (A.11)$$

From production-based perspectives, the estimation of net carbon leakage (NCL) from regions i to other regions based on the allocation for CLR is shown as equation (A.12).

$$NCL_i^{\text{pro}} = \sum_{j\neq i} CLR_{i(j)} - \sum_{j\neq i} CLR_{j(i)} \quad (A.12)$$

From consumption-based perspectives, the estimation is shown as equation (A.13).

$$NCL_i^{\text{con}} = \sum_{j\neq i} CLR_{j(i)} - \sum_{j\neq i} \text{CLR}_{i(j)} \quad (A.13)$$

Text S3 Detailed projects of carbon peaking pathway by LEAP model

The LEAP (Long-range Energy Alternatives Planning system) model bottom-up approach integrates factors such as population, economy, and technology. It predicts future carbon emission trends and peak pathways under different scenarios of economic growth, energy and industrial structure adjustments, and technological progress [1, 2]. Compared to other methods like multiple regression, the LEAP model offers structural stability and flexible data input, allowing adjustments based on research needs and data availability. Focusing on the Guangdong–Hong Kong–Macao Greater Bay Area (GBA), LEAP model is utilized to analyze final energy demand and relevant carbon emissions. The final energy demand is categorized into industries such as electricity, heating and gas, manufacturing, services, agriculture, transportation, construction and mining, considering fossil fuels and other energy sources.

By integrating with emission factor method to estimate carbon emissions of GBA and its cities, based on the sector-specific activity data and emission factors of various emission factors, the estimation is shown by equations (A.14) and (A.15).

$$PBE = C'_{\mathrm{P}} = C'_{\mathrm{F}} \tag{A.14}$$

$$C'_{\mathrm{f}} = \sum_{\mathrm{y}} \sum_{\mathrm{g}} A'_r \times E'_r \times \theta_{rs} \times E'_p \tag{A.15}$$

where C'_{P} and C'_{F} represents production-based carbon emissions and emissions generated by final energy demand; A'_r denotes the economic activity level of the sector r, while E'_r represents the energy intensity of sector r, θ_{rp} represents the proportion of p-type energy demand on the total of sector r, E'_p represents the associated emission factor of p-type energy carbon emissions.

Text S4 Introduction of BEF and the estimation of HCS

The biomass of forest stands was estimated applying the data of stock volume, basic timber density and biomass expansion factor of the vegetation [3, 4]. Biomass expansion factor (BEF) can estimate the carbon sequestration of specific vegetation based on its type, species, age, stock volume, growth rate, and some local characteristics, which can help us to more precisely distinguish the tradable forest carbon sink [4, 5]. This method has been widely used and recommended to estimate the biomass carbon stocks by the researchers and governors. There is considerable research which applied to calculate the forest carbon sequestrations on Chinese land [6-8]. It was also utilized by "The people's republic of China National Greenhouse Gas Inventory" and "IPCC Guidelines for National Greenhouse Gas Inventories". With the data of national forest resources inventories, the forest biomass and carbon stock can be accurately measured. Based on "Methodology of forest management carbon sequestration project", only the carbon sequestrations of young and middle age arbor forest can be traded [9, 10], in line with international standard [11]. Numerous previous studies also indicated that carbon absorption of natural forest cannot be ignored. The regrowth of natural forest carbon sequestration is the important strategy for reducing CO_2 emission and alleviating climate change [12]. In addition, carbon sequestration can also provide large economic benefits to undeveloped areas, especially in China [13]. In addition, this method was also utilized by "The people's republic of China National Greenhouse Gas Inventory" and "IPCC Guidelines for National Greenhouse Gas Inventories". Combining with "Chinese National Greenhouse Gas Inventory", we can even consider the local characteristics of each province in BEF calculation. It should be noted that carbon content rate of biomass was usually assumed to be 0.5 [14, 15]. However, the true value varies slightly for different types of vegetation and regions. Here the data carbon content rate of biomass of young and middle age arbor forest in Guangdong were collected and classified by "Chinese National Greenhouse Gas Inventory" in our study.

Here human-related forest carbon sink (HCS) is hypothesized to be equivalent to forest carbon sink with potential for carbon trading. According to the "Forest

Management Carbon Sequestration Methodology" of the China Certified Emission Reduction (CCER) project, only artificial young and middle-aged forests can be used for trading [16]. However, natural forest, given their considerable potential for carbon storage, are integral to the attainment of carbon neutrality [12, 17, 18]. As a result, "Methodology of carbon sequestration project for natural secondary forest management" has begun to be developed, grounded in the theoretical frameworks prevalent in domestic carbon trading markets. This development underscores the increasingly pivotal role that natural forest resources may play in forest carbon trading, highlighting their significant potential within future carbon markets.

Text S5 Projects of future HCS

Combining provincial forest volume with the land-use remote sensing monitoring data (1980–2015) from the Chinese Academy of Sciences allows us to access how GBA 11 cities varied for the years 2005, 2010, 2015, and 2020, thus providing the value of forest carbon sink in each city for these years. Following the same method, the forest carbon sink in each district or city from 2005 to were obtained. In terms of forecasting, based on the 14th relevant plans for forestry development of Guangdong and 11 cities, forest volume targets were obtained for Guangzhou, Shenzhen, and Zhaoqing in 2025 and 2030. For cities where pertinent information was not available, this study combined the historical growth rate with provincial growth target of forest volume to project future volumes. It should be emphasized that under the existing definition of carbon neutrality, carbon sink offset is achieved by purchasing carbon reduction allowances. In the prevailing guidelines for carbon neutrality of China, such as the “Implementation guidelines for carbon neutralization of large-scale activities”, carbon neutrality refers to the process of counterbalancing the greenhouse gas emissions from large-scale events through carbon credits, carbon quotas purchasing or by creating fresh forestry projects to generate carbon sinks. Hence, those forest carbon sink with trading capabilities were considered as human-related forest carbon sink in our study.

Text S6 Study area

Taking the GBA as study area, the urban agglomeration was chosen to explore mechanisms for allocating urban carbon mitigation responsibilities. GBA consists of nine cities and two administrative regions, Hong Kong and Macao. The total area is approximately 56,000 square kilometers, with a population of 70 million and an economic output exceeding 10 trillion yuan. Both of these metrics surpass those of the Yangtze River Delta and Beijing–Tianjin and Hebei, demonstrating significant strategic relevance in the national transition towards high-quality development and green transformation. Over the past decades, GBA has maintained an annual rate of GDP growth at around 8%. However, alongside rapid economic development, energy consumption and corresponding carbon emissions in this area have been on sharp rise. As an essential pillar of the Belt and Road Initiative, GBA is strategically located along the southeastern coast of China. It lies proximate to the main international shipping routes, thereby offering a logistical advantage that allows faster access to Southeast Asia, South Asia, the Middle East, and Europe. Thus, it can be regarded as a global hub for transportation, logistics, and also a center for international cultural exchange. In the context of China's contemporary dual circulation strategy, GBA is set to fully exploit the function and role of the urban agglomeration in national export expansion, while simultaneously participating in international economic cooperation and competition at an elevated level. Over the past decade, the ports in GBA have drawn extensive international commercial activity with advantageous geographical locations and favorable trade conditions. In particular, cities within the Pearl River Delta (PRD), such as Dongguan and Foshan with robust manufacturing foundations and strong export capabilities, have attracted a multitude of both international and domestic enterprises to establish their presence in the area, fueling vibrancy for trade-related activities. Nonetheless, due to the distinct developmental environments, conditions, and specific implementary policies across various regions, considerable economic and social disparities exist among the cities. Moreover, the inland area within GBA is rich in forest resources, with Guangdong Province boasting a forest coverage rate as high as 58.59%.

Text S7 Production-based carbon peaking pathways for the 11 cities of GBA

This study forecast for production-based carbon emissions from 2020 to 2035 in GBA is depicted in Fig. S8. All eleven cities within the area can ideally reach their carbon emission peak before 2030 under a majority of circumstances. Taking peaking times, maximum values, and emission levels into consideration amongst feasible peaking scenarios, the enhanced energy and climate policy (EECP) scenario appears to be the most favorable for most cities. Among these scenarios, the earliest peaking years (before 2025) appeared in EECP for most cities which can also be regarded as the suitable pathway for economically developed cities, such as Guangzhou and Shenzhen. However, for some undeveloped cities with great potential of economic development and abundant forest resources, the SCEP with steady GDP growth is appropriately designated as the recommended future developmental pathway.

Guangzhou

As an essential global business hub and transport junction in China, carbon emissions in Guangzhou are estimated to peak between 99.5 million and 120.56 million tons by 2030. The city’s crucial role in GBA makes its low-carbon strategies important for regional integration. To meet the ambitious target by 2030, the city needs to implement more stringent energy policies and foster rapid transformations in energy structure. Guangzhou thrives in energy-saving renovation technology, enforcing low-carbon policies, and supervising high-energy-consuming firms. With further efforts in advancing green transformations, promoting eco-friendly lifestyles, and increasing resource efficiency, Guangzhou is poised to progress towards a green, low-carbon, and cyclic future as outlined in the city’s 14th Five-Year Plan for National Economy and Social Development and the Visionary Objectives for 2035.

Hong Kong

As the third largest financial hub in the world, Hong Kong plays an essential role on play in international trade, shipping, and innovation technology. Collectively, these industries make Hong Kong greatly vulnerable to external political environments shift. The carbon emissions of the city could peak at 33.17–37.48 million tons by 2030, given conditions outside of the scenarios with robust GDP growth. With more stringent

carbon mitigation policies, a lower steady-growth rate might be realized by 2021. The progress of the city towards decarbonization, energy efficiency, green construction, and transportation has shown promising signs in achieving Paris Agreement targets and reducing fossil energy consumption.

Macao

Operating as an international free port for tourism and leisure, prosperous economy of Macao heavily relies on light industry, casinos, and the hospitality sector. Its carbon emissions are projected to peak between 1.37 and 1.56 million tons under most scenarios by 2026, but have the potential to reach its peak earliest by 2021 in the scenario of EECP with steady GDP growth through optimizing industry structure and reducing energy intensity. In light of its higher per capita GDP and lower energy consumption in line with other developing nations, Macao demonstrates how economic development can be independent of energy consumption growth, focusing on intensive, low-carbon energy use. A gradual shift to an electrical transport system and cleaner power production under Stated energy and climate policy scenario (SECP) will decrease fossil energy usage, positioning this city favorably for carbon peaking.

Shenzhen

Shenzhen, a vital city in the GBA, is a globally recognized as a science and technology innovation hub and national logistics center. This highlights its economic strengths in culture, technology, logistics, and finance. Carbon emissions are forecast to peak between 38.76 million and 47.39 million tons by 2030 under all scenarios except high economic growth, under which the peak will be reached later. The quickest peaking scenario is the EECP with conservative GDP growth, anticipated by 2024. As a first-tier city and regional development engine, Shenzhen is recommended to hasten its industrial stronghold transformation and consider the scenario of EECP with conservative GDP growth for rational emission reduction. Balancing fast socio-economic development and carbon peaking could mean adopting the steady GDP growth, which implies a peak by 2029. Major shifts in economic development will notably affect carbon emissions, increasing Shenzhen’s peaking emissions and thus pushing back its peaking times.

Foshan

As a key economic hub in Pearl River Delta (PRD), Foshan plays a significant role in China's manufacturing sector. Carbon emissions are projected to peak between 37.81 million and 41.59 million tons by 2029 under selected scenarios. Shifting Foshan's industrial focus from electricity, heat, gas, and manufacturing (accounting for 55.3%) with steady GDP growth allows a high growth rate alongside carbon peaking. Its 14th Five-Year Plan encourages a higher level of interstate connectivity within the Greater Bay Area and fosters the development of a modern industrial system.

Dongguan

As a thriving city in Guangdong Province, Dongguan is recognized for its robust manufacturing sector, which includes eight pillar industries. Carbon emissions are predicted to peak between 56.84 and 62.27 million tons. With the exception of one, all scenarios anticipate the carbon peak before 2030, including as early as 2020 under certain low-carbon growth strategies. Aiming for energy transition, Dongguan may choose higher growth and transformation buffer, the scenario of SCEP with robust GDP growth. With a focus on manufacturing, this city can achieve substantial carbon reduction by improving industrial and energy structures as well as deploying low-carbon technologies. Its 14th Five-Year Plan aims for high-quality development, digitizing the industry, advancing the local brand, and fostering greener manufacturing practices.

Huizhou

Huizhou, a Type II city in Guangdong recognized as the “Gateway to Eastern Guangdong”, bases its economy primarily on electronic information and petrochemicals manufacturing. The carbon emissions of this city, ranging from 30.71 to 36.58 million tons anticipate their peak prior to 2030 under the scenario of EECP with conservative GDP growth but post-2029 under other scenarios. As a second-tier city, Huizhou's potential for economic development is vast due to high business energy intensity and low use efficiency, despite being dominated by traditional industrial manufacturing strategies. Green transformation and technological upgrading should parallel rapid economic and social growth to help achieve Huizhou's predetermined

carbon peak.

Jiangmen

Jiangmen, a node city in GBA, hosts agricultural processing, food manufacturing, and electronic equipment manufacturing as its pillar industries. Carbon emissions peak of this city between 20.76 and 26.65 million tons. Almost all scenarios can achieve carbon peaking by 2027. To promote green growth and quicken peaking attainment, intensified the scenario of SECP with robust GDP growth should be implemented given Jiangmen's established new energy, and ecological carbon sequestration industries. As the region expands in photovoltaic projects and clean energy proportion, rapid progression towards carbon peaking goal is anticipated.

Zhongshan

As a second-tier city, Zhongshan stands as a national emblem of Guangdong culture and heritage. Four core sectors, namely advanced electronic information, high-quality equipment manufacturing, biomedicine and health, and smart home appliances underpin the economy. Carbon emissions in Zhongshan oscillate between 8.43 and 8.73 million tons annually with multiple potential peak paths available. Under the SECP with robust GDP growth scenario, socioeconomic development can be prioritized, providing space for economic growth. The city has achieved public transport electrification, renewable energy system enablement, efficient energy supply and reduced energy intensity, which is anticipated to be achieved by 2025.

Zhuhai

The unique geographical position connects Zhuhai with Hong Kong and Macao. The core industries of Zhuhai include electronic information and biomedicine. Carbon emissions hover between 17.79 and 18.71 million tons. Attaining emission peak by 2030 appears feasible except under robust GDP growth scenarios, with the most rapid progress anticipated under EECP with conservative GDP growth. Transitioning towards the service industry aids early carbon peaking achievement through green industrial development.

Zhaoqing

As a central-western transport hub in Guangdong, Zhaoqing gradually shifted

focus to manufacturing industry. Carbon emission peak of Zhaoqing lies between 22.22 and 26.07 million tons, achieving carbon peaking before 2030 under most scenarios but BAU and SECP with robust GDP growth. The earliest peak comes under EECP with conservative GDP growth. Despite being a third-tier city, vigorous environmental strategies may be helpful to attain carbon peaking. Economic growth here primarily shows an industrial, high-energy-consumption pattern, which delays carbon peaking compared to economically advanced areas in the Greater Bay Area. The 14th Five-Year Plan advocates to accelerate the development of sectors like battery-supported car industry and high-end construction, aiming to revitalize rural areas such as Zhaoqing in GBA.

Text S8 Consumption-based carbon predictions for GBA

Fig. S9 displays consumption-based carbon emissions under various scenarios in GBA. Excluding robust GDP growth scenario of Hong Kong, 2025 consumption-based emissions in the remaining cities outweigh those of 2030, implying potential for achieving carbon peaking by 2030. Across the developmental scenarios from conservative GDP growth to robust GDP growth, the consumption-based carbon footprint decreases by 9–12%, shifting from 715–764 million tons in 2025 to 630–697 million tons in 2030. Individually, Hong Kong maintains the highest emissions, accounting for 41–42% of the regional emissions. Guangzhou and Shenzhen follow, but show reductions in emission levels by 28–32% and 11–16% respectively from 2020 to 2025, indicating possible realizations of consumption-based emission reductions in the 14th Five-Year Plan period.

It also indicates that Guangzhou and Shenzhen could reach peaking within 2020-2025. For Zhuhai, Foshan, Jiangmen, Zhaoqing and Huizhou, their carbon emissions of 2025 will equal to the amount of 2020, suggesting they also reach the peaking within 2020-2025. Dongguan and Zhongshan, however, show rising emissions by 28–36% and 6–12%, respectively during the same period. Moreover, this study reveals consumption-based emission growth being non-linear under different scenarios with increasing economic growth rate due to carbon intensity reduction target impact. For example, under steady GDP growth scenario, the consumption-based emission of Hong Kong in 2030 is 7.4% greater than that under conservative GDP growth scenario, but 8.3% less than that under robust GDP growth scenario. Hence, economic growth is a driving factor for emission growth in GBA, emphasizing the need for balanced economic development, improved regional energy structure, and technology advances to reduce carbon emission intensity.

B. Supplementary Figures

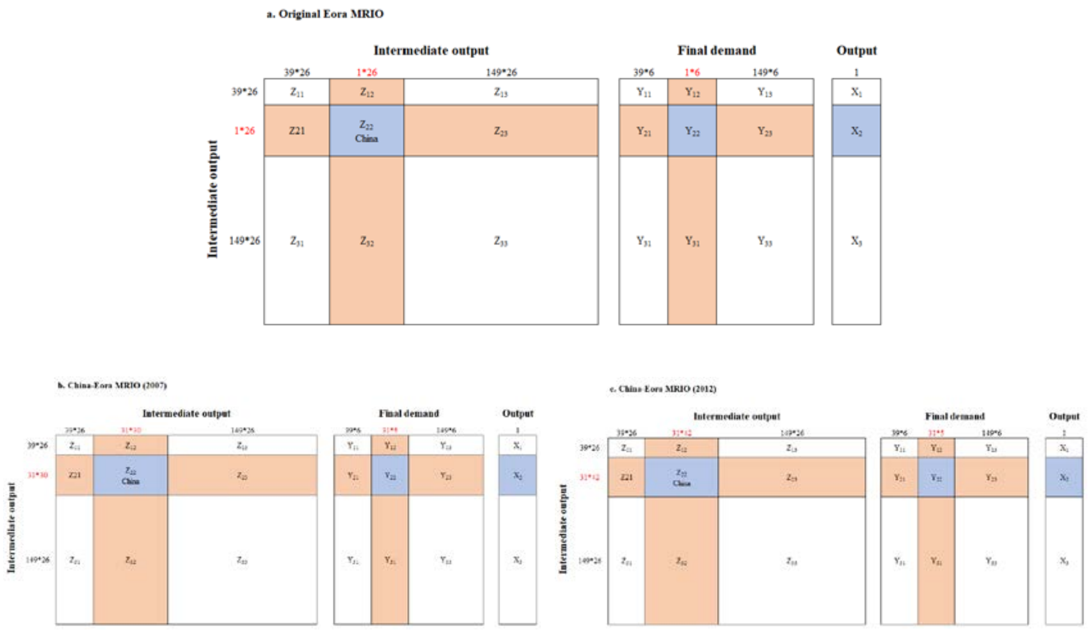

Fig. S1. Establishment of nested MRIO table. This figure indicates the dimensions for the matrixes. Matrixes in blue are domestic structure of China's MRIO, matric in orange are China's exports and imports, and matric in white do not change. In our study, we build a new China MRIO through combining Chinese MRIO for 31 provinces and 30 and 42 sectors with Eora MRIO for 140 countries and 26 sectors. The specific method for linking 30 and 42 sectors of China’s MRIO and 26 sectors of Eora are referenced by existed study [19]. At first, matrixes for China's domestic structure in Eora global MRIO directly (in blue). Z_{12} and Z_{32} are China's imports for intermediate use, Z_{21} and Z_{23} are China's exports for intermediate use, Z_{22} is China's domestic production structure, y_{12} and y_{32} are China's imports for final use, y_{21} and y_{23} are China's exports for final use. X_2 is China’s total output. In addition, we also assumed that international imports and exports of each sector in a China's province are distributed among all foreign countries in the same proportion as China's exports and imports of the sector when calculating the matrix for China's international imports and exports (in orange). Besides, matrixes for other countries do not change (in white).

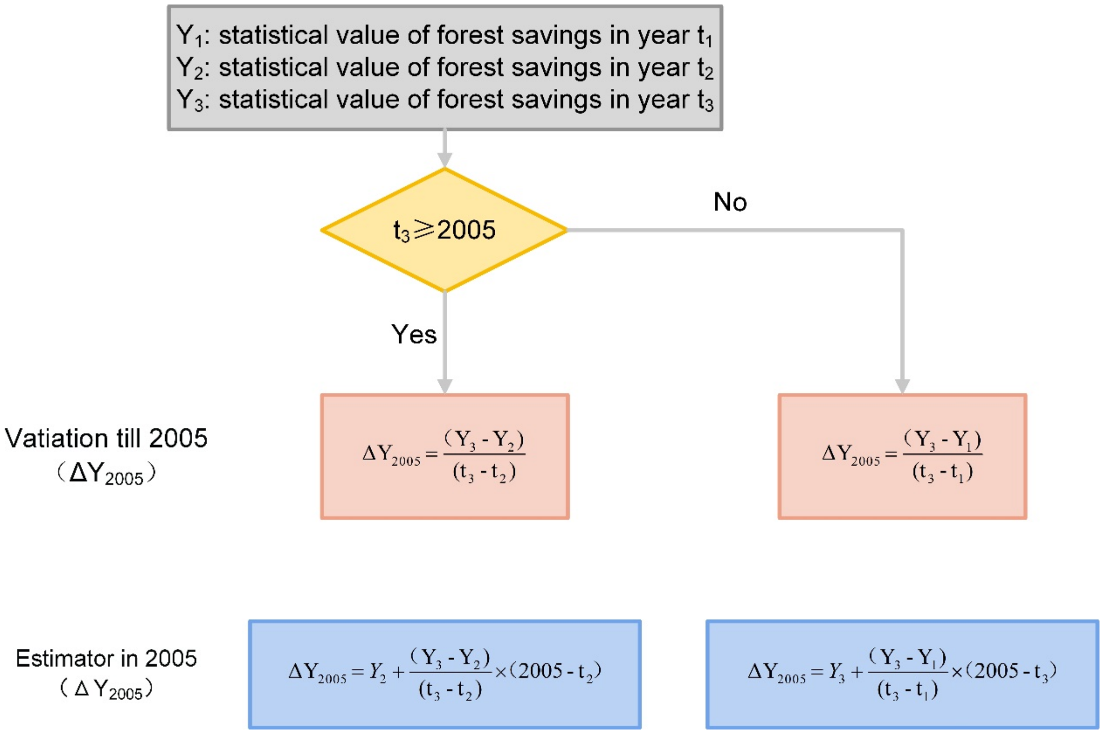

Fig. S2. Interpolation and extrapolation method for forecasting changes of forest carbon sink

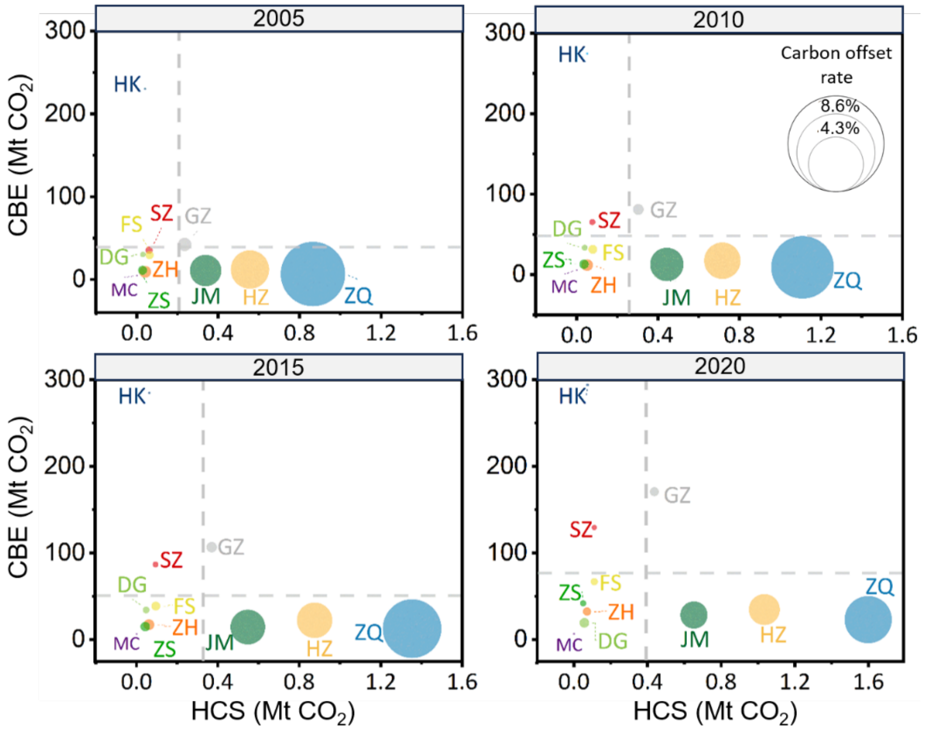

Fig. S3. Dynamic distributions of consumption-based CO_2 emissions (CBE) and human-related forest carbon sink (HCS) during 2005–2020. The cities are abbreviated as follows: Guangzhou (GZ), Shenzhen (SZ), Huizhou (HZ), Jiangmen (JM), Foshan (FS), Zhaoqing (ZQ), Zhongshan

(ZS), Zhuhai (ZH), Dongguan (DG), Hong Kong (HK), and Macao (MC).

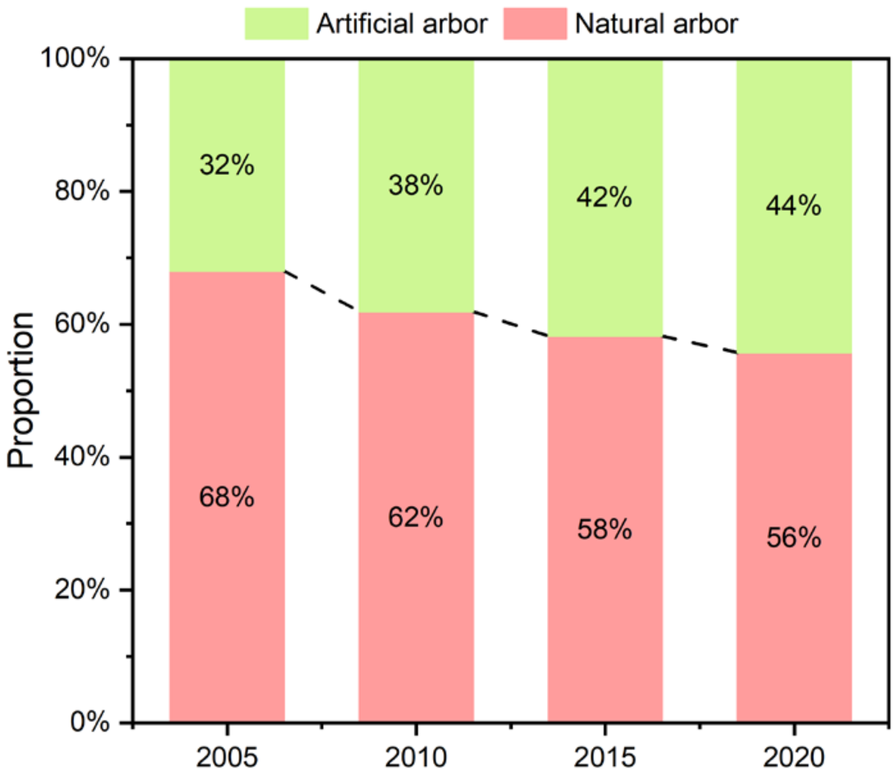

Fig. S4. Shift in proportions of artificial and natural arbors in human-related forest carbon sink between 2005 and 2020.

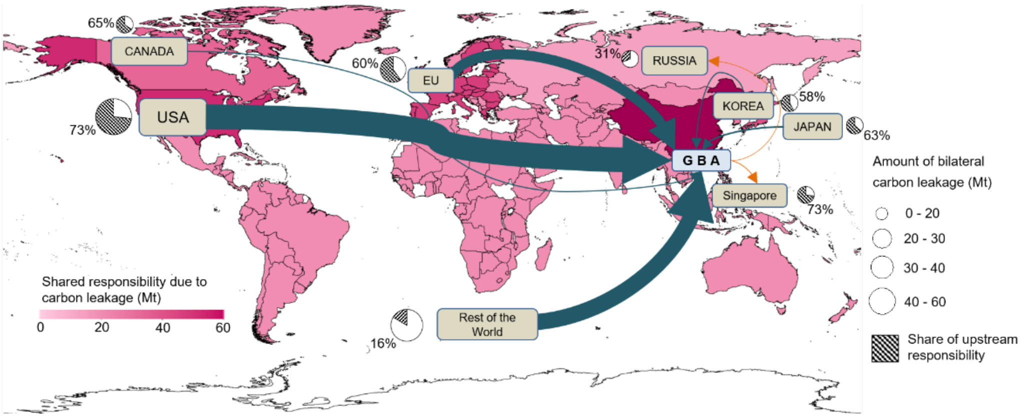

Fig. S5. Allocation of CMRs between GBA and other regions due to carbon leakage. The flows denote the CMRs attributed to GBA due to carbon leakage. Blue and orange arrows show responsibility resulting from imports and exports, respectively.

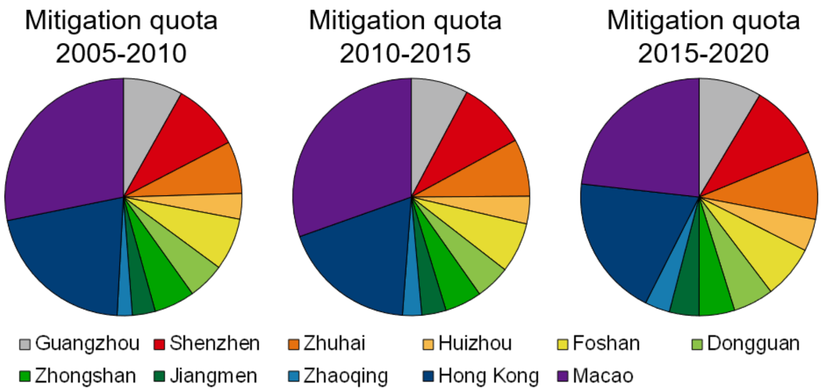

Fig. S6. Cities' shares of required mitigation quota in GBA under the principle of economic capability (GDP per capita) during the three historical periods (2005–2010, 2010–2015 and 2015–2020).

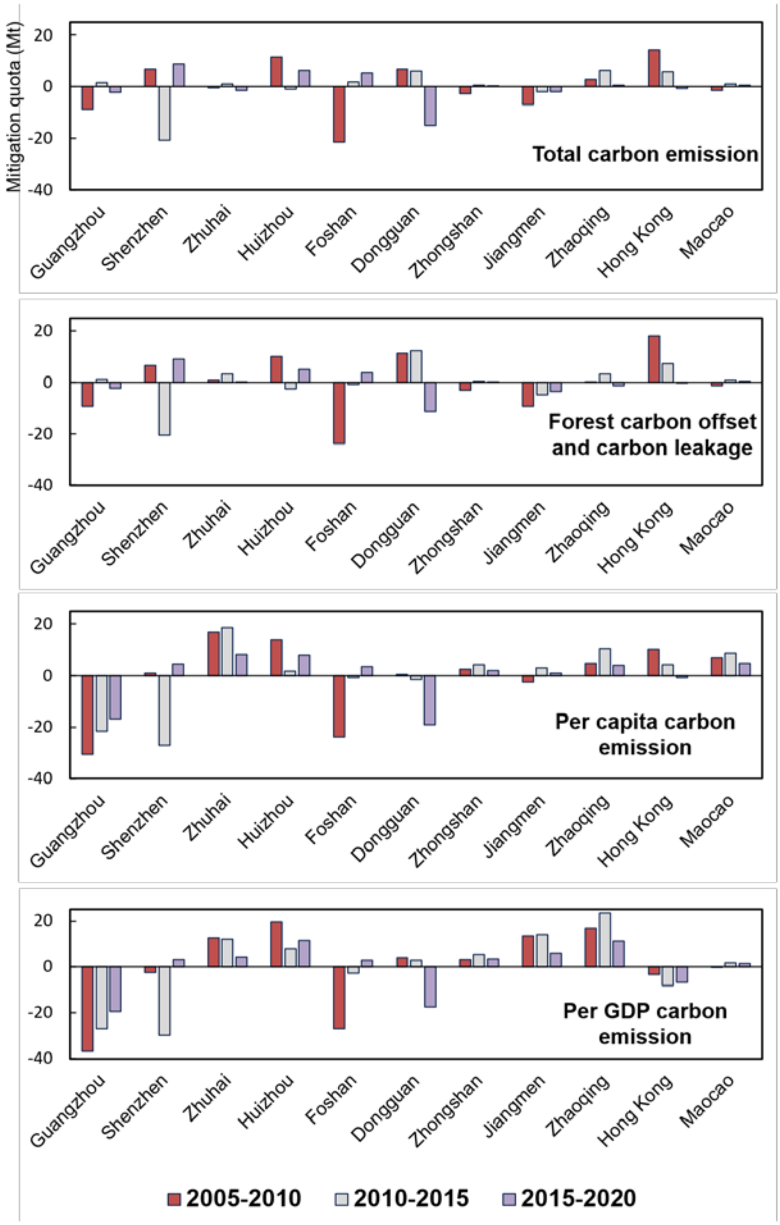

Fig. S7. Mitigation quota difference (MD) in GBA cities during 2005–2020 under various metrics of emission.

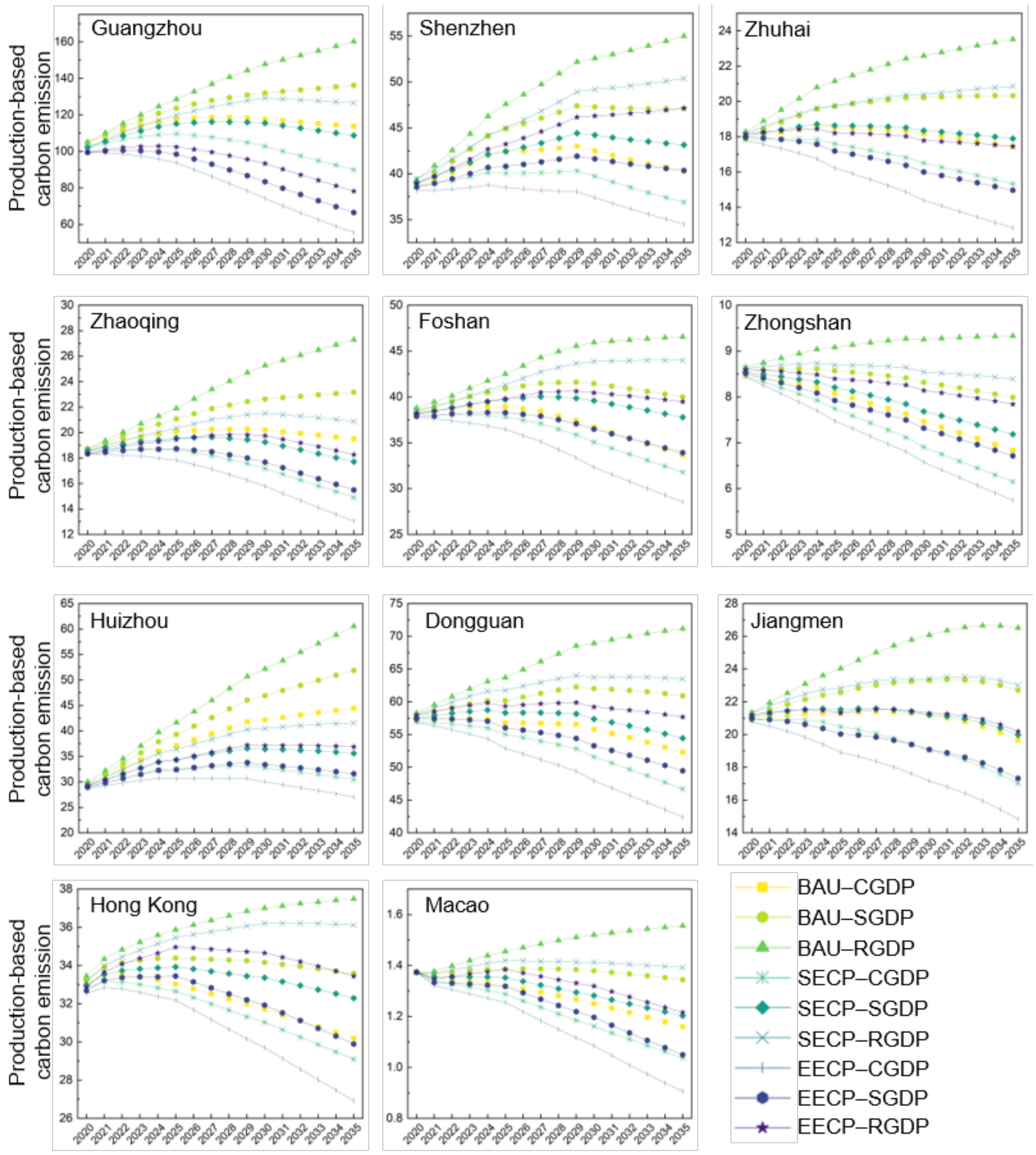

Fig. S8. Production-based carbon emission predictions for the cities of Guangdong-Hong Kong-Macao Greater Bay Area (GBA) under different scenarios. BAU, SECP and EECP represent business as usual, stated energy and climate policy scenario, and enhanced energy and climate policy scenario. CGDP, SGDP and RGDP represent conservative GDP growth, steady GDP growth, and robust GDP growth.

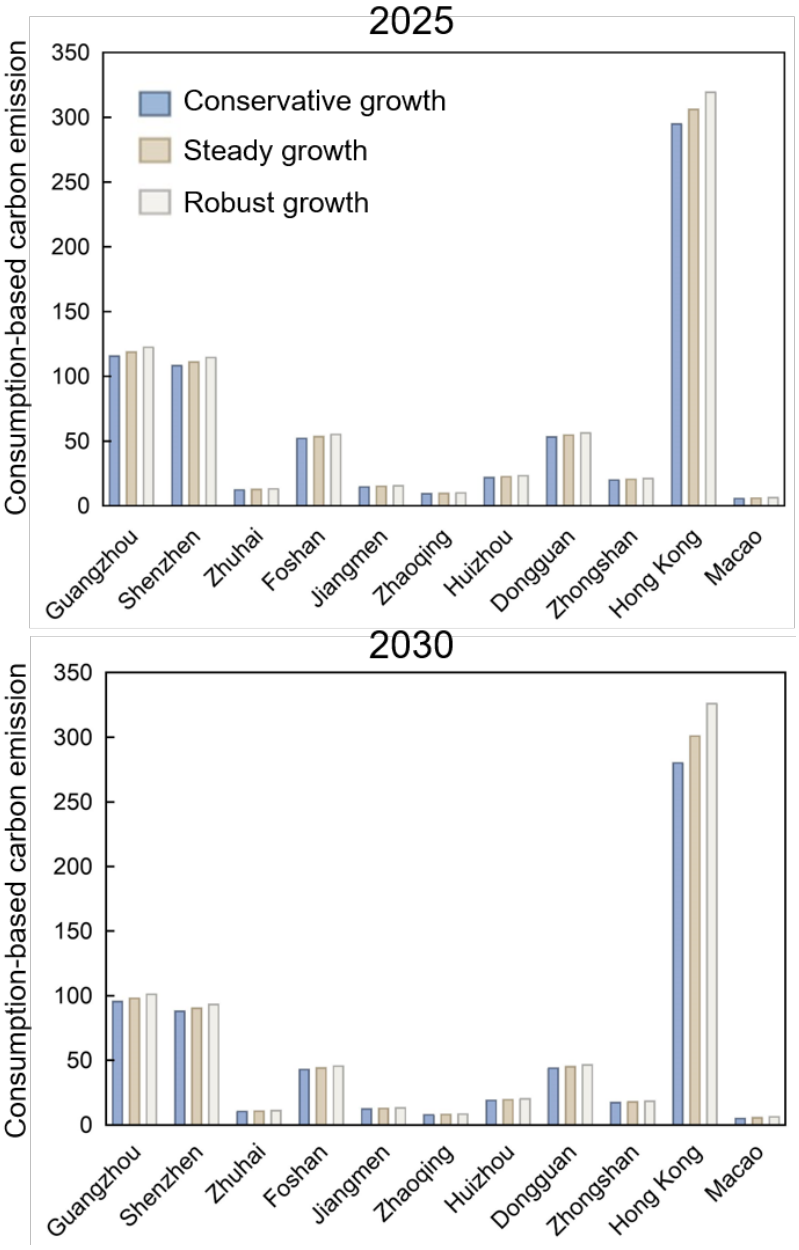

Fig. S9. Projection of Consumption-based carbon emission for the cities of Guangdong-Hong Kong-Macao Greater Bay Area (GBA) under the developmental scenarios.

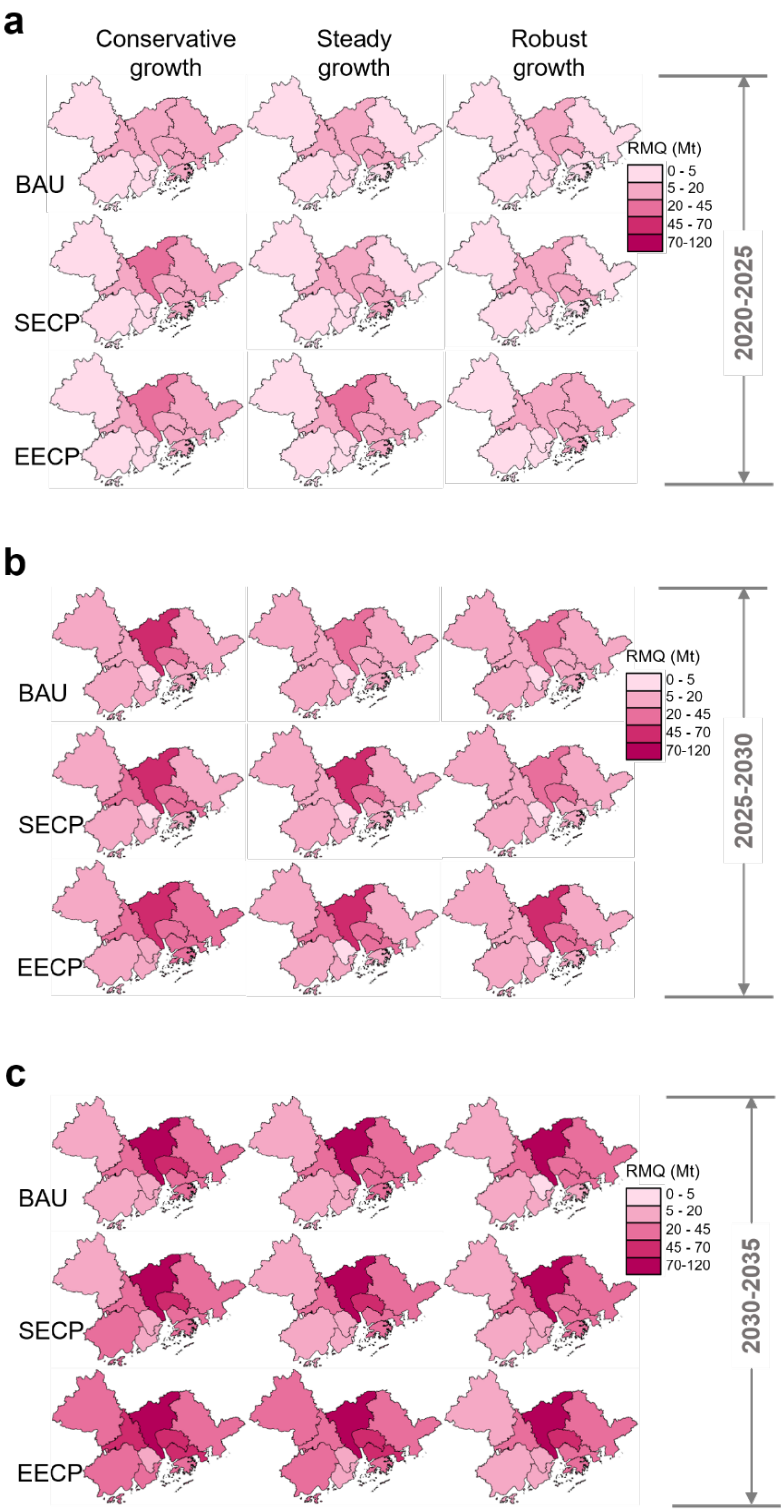

Fig. S10. Allocation of cumulative required mitigation quota from production-based perspective (a) from 2020 to 2025, (b) from 2025 to 2030, and (c) from 2030 to 2035. BAU (business as usual), SECP (stated energy and climate policy scenario), and EECP (enhanced energy and climate policy scenario).

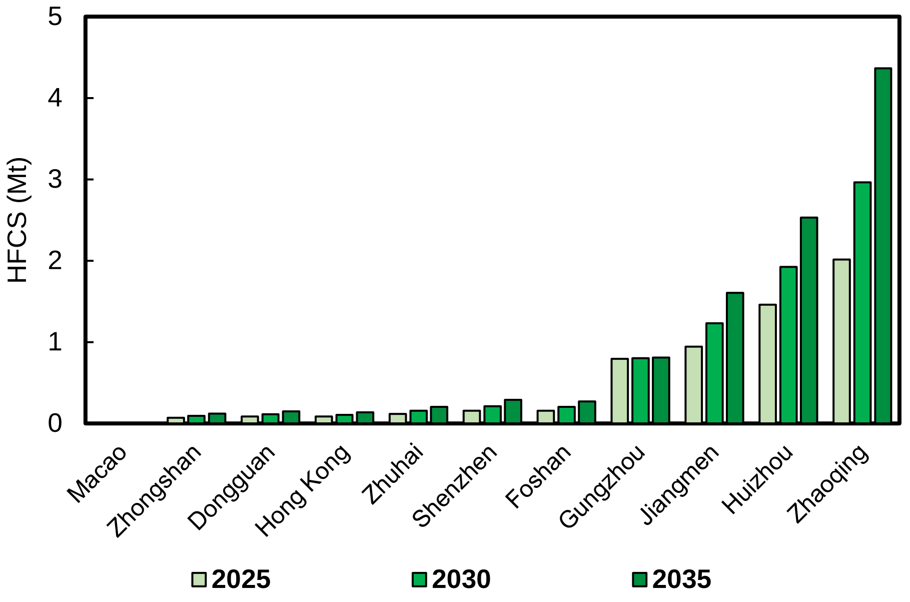

Fig. S11. Projected human-related forest carbon sink (HCS) for the cities of GBA.

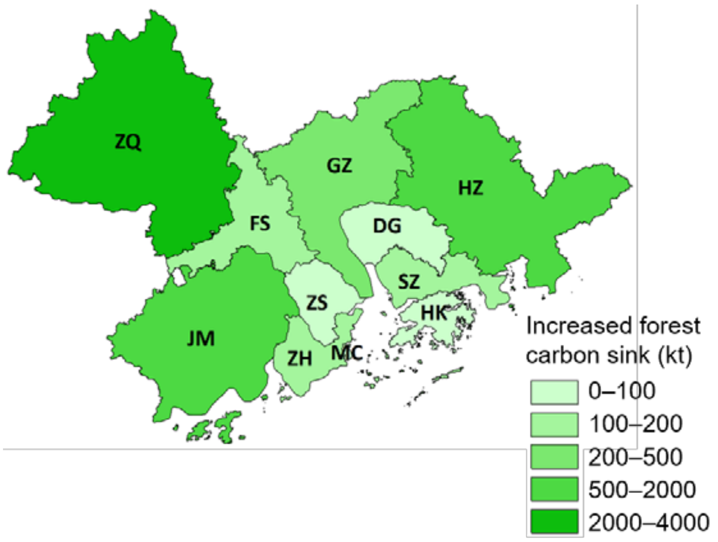

Fig. S12. Increased forest carbon sink in GBA cities during 2020-2035. The cities are abbreviated as follows: Guangzhou (GZ), Shenzhen (SZ), Huizhou (HZ), Jiangmen (JM), Foshan (FS), Zhaoqing (ZQ), Zhongshan (ZS), Zhuhai (ZH), Dongguan (DG), Hong Kong (HK), and Macao (MC).

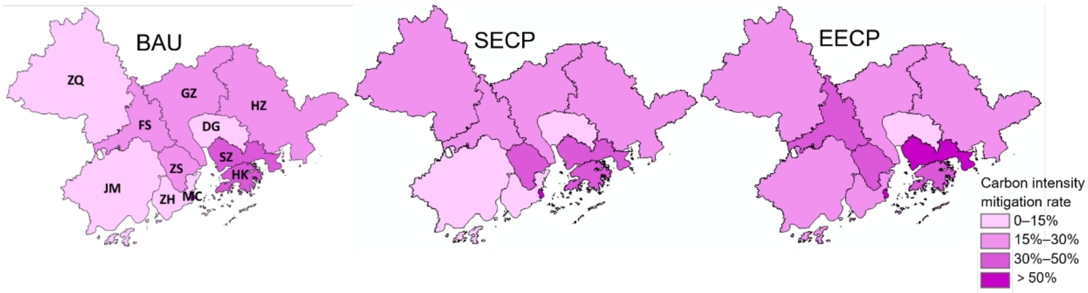

Fig. S13. Mitigated carbon intensity based on the current plans in GBA cities during 2020-2035 under the scenarios of steady growth. Note: BAU, SECP and EECP represent business as usual, stated energy and climate policy scenario and enhanced energy and climate policy scenario.

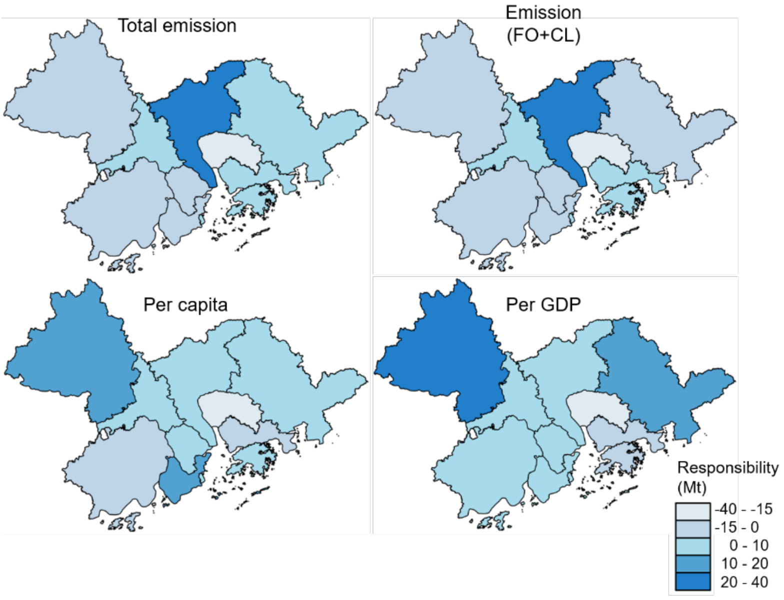

Fig. S14. Mitigation quota difference (MD) of cities in Guangdong-Hong Kong-Macao Greater Bay Area (GBA) during 2020-2035 under SECP (stated energy and climate policy) scenario with steady economic growth. FO and CL represent forest offset and carbon leakage, respectively.

C. Supplementary Tables

Table S1. Future scenario setting for carbon mitigation in Guangdong-Hong Kong-Macao Greater Bay Area (GBA)

Scenario type	Business as usual	Stated energy and climate policy scenario	Enhanced energy and climate policy scenario
**Scenario note**	Continuation of policies from the 13th Five-Year Plan and before, with reference to historical trends in industrial structure and energy demand indicators	Prioritize low-carbon development and make certain shifts in the social model to ensure that national and provincial emissions reduction targets are met	Implementing more stringent emission reduction measures, exceeding the established emission reduction targets and moving as close as possible to the level of developed country regions
**Description**	Industrial structure, energy structure and energy intensity continue to develop in accordance with the status quo and trends during the 13th Five-Year Plan period	The industrial structure and energy structure have been gradually optimized, with the proportion of high-energy-consuming industries such as electricity, heat and gas gradually declining, the proportion of high-tech industries and service industries rising, the proportion of coal and oil in energy consumption steadily declining, the proportion of natural gas and electricity rising, and the intensity of energy steadily decreasing	The industrial structure is further transformed and upgraded, the decline of high-energy-consuming industries increases, high-tech industries and service industries develop faster, coal consumption is replaced by natural gas or other clean energy sources at a faster rate, and the decline of energy intensity increases

Table S2. Future scenario setting for socioeconomic development in Guangdong-Hong Kong-Macao Greater Bay Area (GBA)

Scenario type	Conservative GDP growth	Steady GDP growth	Robust GDP growth
**Scenario note**	Indicators such as GDP per capita and population size have lower values than the baseline scenario	Baseline scenario based on historical trends and 14th Five-Year Plan policy settings	Higher level of economic development than the baseline scenario, i.e., higher values of indicators such as GDP per capita, population size, etc., than the baseline scenario
**Description**	a. With the implementation of the three-child policy, coupled with the vibrant economy of GBA, the population is still on an increasing trend, but the growth rate has decreased. Due to the objective of accelerating the structural transformation of the economy, prompting better adaptation to the new normal in the future, and promoting higher quality development, the rate of economic growth will decrease as the total economy continues to expand<br>b. Considering common prosperity and encouraging emission reduction in relatively backward regions at a smaller economic cost, a higher economic growth rate will be set in economically relatively backward regions<br>c. Considering the forestry carbon trading program, a higher economic growth rate will be set in the relatively backward regions with richer forest resources, which will promote the synergistic development of the region and reduce the economic differences between regions, thus promoting the realization of the goal of common prosperity		

Table S3. Detailed information of data source

Data category	Sources
Socioeconomic factors of each city in GBA (population, Gross Domestic Product (GDP), and expenditure levels related to consumption)	Guangdong Statistical Yearbook
Direct energy consumption, production-based carbon emission data across diverse departments within the nine cities of PRD	China Emission Accounts & Datasets (CEADs) database
Production-based carbon emission by industries in Hong Kong, Macao and foreign regions	PRIMAPHIST database in EORA
China's multi-regional input-output tables in 2007, 2012 and 2017	National Bureau of Statistics of China
Global multi-regional input-output tables in 2007, 2012 and 2017	EORA database
Area and stock volume of middle-aged and young forests in the artificial and natural arbor forests of each PRD city	The 6th to the 9th National Forest Resources Inventory Bulletin
Volume of natural and artificial young and middle-aged forests	The 7th and 9th national forest resources inventories
Growth rates of plantations and native forest biomass, basic wood density, biomass expansion factors, ratio of underground biomass to aboveground biomass, along with carbon content	The people's republic of China National Greenhouse Gas Inventory

SI References

[1] J. Dong, C. Li, Q. Wang, Decomposition of carbon emission and its decoupling analysis and prediction with economic development: A case study of industrial sectors in Henan Province, J. Clean. Prod. 321 (2021) 129019.

[2] J. Jiang, B. Ye, S. Shao, N. Zhou, D. Wang, Z. Zeng, J. Liu, Two-tier synergic governance of greenhouse gas emissions and air pollution in China's megacity, Shenzhen: Impact evaluation and policy implication, Environ. Sci. Technol. 55 (2021) 7225-7236.

[3] J.-Y. Fang, G.G. Wang, G.-H. Liu, S.-L. Xu, Forest biomass of china: An estimate based on the biomass–volume relationship, Ecol, Appl, 8 (1998) 1084-1091.

[4] J. Fang, A. Chen, C. Peng, S. Zhao, L. Ci, Changes in forest biomass carbon storage in China between 1949 and 1998, Science 292 (2001) 2320-2322.

[5] M. Zhao, J. Yang, N. Zhao, X. Xiao, T. Yue, J.P. Wilson, Estimation of the relative contributions of forest areal expansion and growth to China's forest stand biomass carbon sequestration from 1977 to 2018, J. Environ. Manage. 300 (2021) 113757.

[6] C. Zhang, W. Ju, J.M. Chen, M. Zan, D. Li, Y. Zhou, X. Wang, China’s forest biomass carbon sink based on seven inventories from 1973 to 2008, Climatic Change 118 (2013) 933-948.

[7] J. Guan, H. Zhou, L. Deng, J. Zhang, S. Du, Forest biomass carbon storage from multiple inventories over the past 30 years in Gansu Province, China: Implications from the age structure of major forest types, J. Forest. Res. 26 (2015) 887-596.

[8] H. Zhang, D. Guan, M. Song, Biomass and carbon storage of Eucalyptus and Acacia plantations in the Pearl River Delta, South China, Forest Ecol. Manage. 277 (2012) 90-97.

[9] X.J. Meng, N.H. He, K. Zhou, Study on measuring carbon sequestration of forest management project —— A case of pinus massoniana stand in Wanzhou Disrict, Chongqing, Resour. Dev. Market. 30 (2014) 444-447.

[10] X.U. Bing, Z.D. Guo, S.L. Amp, Biomass carbon stocks in China's forests between 2000 and 2050: A prediction based on forest biomass-age relationships, Sci. China Life Sci. 53 (2010) 776-783.

[11] B. Manley, P. Maclaren, Potential impact of carbon trading on forest management in New Zealand, Forest Policy Econ. 24 (2012) 35-40.

[12] S.C. Cook-Patton, S.M. Leavitt, D. Gibbs, N.L. Harris, K. Lister, K.J. Anderson-Teixeira, R.D. Briggs, R.L. Chazdon, T.W. Crowther, P.W. Ellis, H.P. Griscom, V. Herrmann, K.D. Holl, R.A. Houghton, C. Larrosa, G. Lomax, R. Lucas, P. Madsen, Y. Malhi, A. Paquette, J.D. Parker, K. Paul, D. Routh, S. Roxburgh, S. Saatchi, J. van den Hoogen, W.S. Walker, C.E. Wheeler, S.A. Wood, L. Xu, B.W. Griscom, Mapping carbon accumulation potential from global natural forest regrowth, Nature 585 (2020) 545-550.

[13] B. Lin, J. Ge, Valued forest carbon sinks: How much emissions abatement costs could be reduced in China, J. Clean. Prod. 224 (2019) 455-464.

[14] J. Fang, Z. Guo, S. Piao, A. Chen, Terrestrial vegetation carbon sinks in China, 1981–2000, Sci. China Ser. D. 50 (2007) 1341-1350.

[15] C. Zhang, W. Ju, J.M. Chen, X. Wang, L. Yang, G. Zheng, Disturbance-induced reduction of biomass carbon sinks of China’s forests in recent years, Environ. Res. Lett. 10 (2015) 114021.

[16] B. Xu, Z. Guo, S. Piao, J. Fang, Biomass carbon stocks in China's forests between 2000 and 2050: A prediction based on forest biomass-age relationships, Sci. China Life Sci. 53 (2010) 776-783.

[17] T.A.M. Pugh, M. Lindeskog, B. Smith, B. Poulter, A. Arneth, V. Haverd, L. Calle, Role of forest

regrowth in global carbon sink dynamics, Proc. Natl. Acad. Sci. U. S. A. 116 (10) (2019) 4382-4387.

[18] W.R. Moomaw, S.A. Masino, E.K. Faison, Intact forests in the United States: Proforestation Mitigates climate change and serves the greatest good, Front. For. Glob. Change 2(2019) 27.

[19] L. Shao, D. Guan, N. Zhang, Y. Shan, G.Q. Chen, Carbon emissions from fossil fuel consumption of Beijing in 2012, Environ. Res. Lett. 11 (2016) 114028.
